# Estimating nonlinear effects of random slopes: A comparison of multilevel structural equation modeling with a two-step, a single-indicator, and a plausible values approach

**DOI:** 10.3758/s13428-024-02462-9

**Published:** 2024-07-25

**Authors:** Sarah Humberg, Simon Grund, Steffen Nestler

**Affiliations:** 1 Department of Psychology, University of Münster, Fliednerstr. 21, 48149 Münster, Germany; 2 University of Hamburg, Hamburg, Germany

**Keywords:** Multilevel structural equation modeling, Nonlinear models, Latent interaction, Intensive longitudinal data, Within-person couplings

## Abstract

Multilevel structural equation modeling (MSEM) is a statistical framework of major relevance for research concerned with people’s intrapersonal dynamics. An application domain that is rapidly gaining relevance is the study of individual differences in the within-person association (WPA) of variables that fluctuate over time. For instance, an individual’s social reactivity – their emotional response to social situations – can be represented as the association between repeated measurements of the individual’s social interaction quantity and momentary well-being. MSEM allows researchers to investigate the associations between WPAs and person-level outcome variables (e.g., life satisfaction) by specifying the WPAs as random slopes in the structural equation on level 1 and using the latent representations of the slopes to predict outcomes on level 2. Here, we are concerned with the case in which a researcher is interested in nonlinear effects of WPAs on person-level outcomes – a U-shaped effect of a WPA, a moderation effect of two WPAs, or an effect of congruence between two WPAs – such that the corresponding MSEM includes latent interactions between random slopes. We evaluate the nonlinear MSEM approach for the three classes of nonlinear effects (U-shaped, moderation, congruence) and compare it with three simpler approaches: a simple two-step approach, a single-indicator approach, and a plausible values approach. We use a simulation study to compare the approaches on accuracy of parameter estimates and inference. We derive recommendations for practice and provide code templates and an illustrative example to help researchers implement the approaches.

Many constructs examined in psychological research are latent, meaning that they are not directly observable and can be measured only indirectly. Random slopes as defined in multilevel models are one kind of latent variable that is rapidly gaining relevance in psychological research. Random slopes can be used to represent within-person associations (WPAs) between variables that fluctuate over time (e.g., Back , 2021; Borsboom & Cramer, 2013; Denissen & Penke, 2008; Fleeson , 2007; Kuper et al. , 2022; Mischel & Shoda, 1995; Neubauer & Schmiedek, 2020; Wrzus, Luong, Wagner & Riediger, 2021) and hence allow researchers to investigate effects of WPAs on person-level psychological outcomes. For example, people’s social reactivity can be operationalized as the WPA between repeated measurements of momentary social interaction frequency and well-being (Kroencke, Harari, Back, & Wagner, 2023; Sun, Harris & Vazire, 2020), stress reactivity as the WPA between momentary stress level and negative affect (Bolger & Schilling, 1991; Hisler, Krizan, DeHart, & Wright, 2020; Wrzus et al., 2021), and both might be predictive of people’s interpersonal adjustment or psychological health.

One possible analytical approach for testing effects of WPAs is multilevel structural equation modeling (MSEM; Asparouhov, Hamaker, & Muthén, 2018; Hamaker, Asparouhov, Brose, Schmiedek, & Muthén, 2018; Liang & Bentler, 2004; McNeish & Hamaker, 2019; Muthén , 1989; Muthén & Asparouhov, 2008; Preacher, Zyphur, & Zhang, 2010). MSEM is an extension of regular, single-level SEM to multiple levels of analysis. It can be used to examine the effects of WPAs on person-level outcomes by specifying the WPAs as random slopes $\beta$ in the structural equation at level 1 and using their latent representation as predictors of a person-level outcome on level 2 (e.g., Brose, Neubauer, & Schmiedek, 2022; Liu & Rhemtulla, 2022; Lüdtke et al. , 2008). Treating the random slopes as latent variables when using them in the model of interest takes their imperfect reliability into account (Asparouhov & Muthén, 2019; Lüdtke et al., 2008). Consequently, previous research has found that, for the case of *linear* effects of random slopes on an outcome, MSEM can yield accurate parameter estimates and inferences even in situations in which the slopes can only be estimated with low reliability (e.g., Liu & Rhemtulla, 2022; Neubauer, Brose, & Schmiedek, 2022).

However, psychological theories often imply *nonlinear* associations between WPAs and other psychological constructs, and it is currently unknown how well MSEM can be applied to test them. Therefore, one aim of the present article is to evaluate an MSEM approach that models nonlinear effects of random slopes. Specifically, we considered three classes of nonlinear hypotheses that are often investigated in psychological studies. First, an (inverse) *U-shape* hypothesis captures the idea that a certain level of a predictor variable minimizes (or maximizes) an outcome (e.g., Cohen, Gunthert, Butler, O’Neill, & Tolpin, 2005; Sternberg , 2003). A U-shaped effect of a WPA $\beta$ (e.g., social reactivity) on a person-level outcome *z* (e.g., life satisfaction) can be investigated with an MSEM that estimates the quadratic effect of $\beta$ on *z* in the model on level 2. Second, a *moderation* hypothesis reflects the idea that the strength of the linear relationship between two variables depends on the value of a third variable (e.g., Cummins , 1989; Sung, Chang, & Liu, 2016). In the context of WPAs, a moderation effect of two WPAs $\beta _1$ and $\beta _2$ (e.g., social reactivity and stress reactivity) can be tested with an MSEM that estimates the effect of the interaction between $\beta _1$ and $\beta _2$ on *z*. Finally, a *congruence* hypothesis posits that the similarity between two psychological variables has a positive (or negative) effect on an outcome (e.g., Higgins , 1996; Kim et al. , 2021; Kristof-Brown, Zimmerman, & Johnson, 2005). Drawing from the literature on testing congruence effects with response surface analysis (RSA; Box & Draper, 1987; Box & Wilson, 1951; Edwards & Parry, 1993), the effect of congruence between two WPAs $\beta _1$ and $\beta _2$ can be investigated with an MSEM that estimates the second-order polynomial effects of the two WPAs (i.e., $z = b_0 + b_1\beta _1 + b_2\beta _2 + b_3\beta _1^2 + b_4\beta _1\beta _2 + b_5 \beta _2^2$).

A second aim of this article is to compare the performance of the MSEM approach with alternative modeling approaches that are easier to implement and estimate. One alternative is a *simple two-step* approach, where the WPAs are first estimated as random slopes in a conventional multilevel analysis and the resulting random effect estimates are used to predict the person-level outcome variable in a nonlinear regression analysis. Two-step approaches are often employed as alternatives to fully latent models (SEMs) for their simplicity, especially when the need to implement latent interaction terms can thereby be avoided (Cortina, Markell-Goldstein, Green, & Chang, 2021). However, they are usually inferior to the latent approach because two-step approaches do not account for the unreliability of the estimated scores (Bollen, 1989; Brose et al., 2022; Cole & Preacher, 2014; Neubauer, Voelkle, Voss, & Mertens, 2020; Westfall & Yarkoni, 2016). A second alternative to MESM is a *single-indicator (SI)* approach (Bollen, 1989; Hayduk, 1987) in which latent variables are defined via single-indicator measurement models for the estimated random slopes $\beta$ and then used as predictors in a single-level SEM. This approach is simpler than a fully latent MSEM approach because it proceeds in two separate steps, while it simultaneously takes the slopes’ unreliability into account. A third alternative to MSEM comprises *plausible values (PV)* approaches, which are commonly used to obtain latent variable scores for secondary analysis in large-scale assessment studies (Mislevy , 1991; see also Asparouhov & Muthén, 2010b; Lüdtke, Robitzsch, & Trautwein, 2018; von Davier, Gonzalez, & Mislevy, 2009; Yang & Seltzer, 2016). In this approach, the individual slopes $\beta$ are regarded as missing data and “filled in” with multiple PVs that are generated on the basis of the observed data and a statistical model. The nonlinear level 2 model is then estimated separately for each set of values, and the results are pooled using Rubin’s rules (Rubin, 1987). To our knowledge, the SI and PV approaches have not been evaluated in the context of testing (linear or nonlinear) effects of random slopes. By comparing the four approaches, we aim to contribute to methodological research on estimating nonlinear functions of random slopes, and we aim to support applied research by guiding the choice of method to test hypotheses about nonlinear effects of WPAs.

In the following, we first explain the MSEM approach and the three alternative approaches (simple two-step, SI, PV) for investigating U-shaped, moderation, and congruence effects of WPAs. We then report the results of a simulation study in which we compared the four approaches on accuracy of estimates and inferences. Finally, we derive recommendations for best practice in testing nonlinear effects of WPAs, illustrate their application with example data from a daily diary study, and discuss possible extensions and future developments. To help researchers implement the approaches in their research, we also provide computer code templates for each method (https://osf.io/xucwf).

## MSEM approach for investigating U-shaped, moderation, and congruence effects of WPAs

MSEM is a statistical framework for modeling relationships between variables on different levels of analysis (e.g., Asparouhov et al. , 2018; Hamaker et al. , 2018; Liang & Bentler, 2004; McNeish & Hamaker, 2019; Muthén , 1989; Muthén & Asparouhov, 2008; Preacher et al. , 2010). In the present context, the levels of analysis are repeated measures on level 1 (L1) that are nested within individuals at level 2 (L2). Conceptually, when the aim is to investigate nonlinear effects of WPAs, the general idea of the MSEM approach is to operationalize an individual’s WPA between two repeatedly measured L1 variables *x* and *y* as their individual slope in predicting *y* from *x*. The slopes are then used to predict the L2 outcome *z* of interest in a respective analysis model (e.g., a quadratic model) on level 2. A key feature of the MSEM framework is that all these components are specified in the same model, so that the parameters are estimated in a single step. Below, we describe the MSEM specifications for the three classes of hypotheses (U-shape, moderation, congruence) in more detail.

### MSEM for a WPA-U-shape hypothesis

As an illustrative example, assume that we are interested in individual differences in people’s social reactivity – the extent to which social interactions positively affect an individual’s well-being (e.g., Kroencke et al. , 2023; Sun et al. , 2020) – and that we expect a U-shaped (or inverse U-shaped) effect of people’s social reactivity on their life satisfaction. To test this *WPA-U-shape hypothesis*, we could have collected repeated measures of the quantity of people’s social interactions (*x*) and their momentary well-being (*y*). Assume that we have collected these L1 variables $x_{it}$ and $y_{it}$ at each of $T_i$ time points ($t \in \{1, ..., T_i\}$) for a total of *P* individuals (L2 units) $i \in \{1, ..., P\}$. In addition, assume that we have assessed the life satisfaction $z_i$ for each individual *i* as the L2 outcome of interest.

In the measurement model of the MSEM that can be used to test the example hypothesis, the L1 predictor *x* (sociality) is decomposed into a person’s latent mean, which varies only between persons ($x_B$) and reflects individual differences in people’s sociality, and a second part that captures the person’s within-person fluctuations across time ($x_W$):

1 $$\begin{aligned} &  \!\!\!\text {Measurement model (latent decomposition):} \nonumber \\  &  x_{it} = x_{Bi} + x_{Wit} \end{aligned}$$

In the structural model, a person *i*’s social reactivity is then modeled as the L1 association $\beta _{yi}$ between the person’s fluctuations in sociality $x_W$ and their momentary well-being *y* (e.g., Brose et al. , 2022; Neubauer et al. , 2020):

2 $$\begin{aligned} &  \!\!\!\text {Structural model -- L1: } \nonumber \\  &  y_{it} = y_{Bi} + \beta _{yi} x_{Wit} + \zeta _{Wyit} \end{aligned}$$

3 $$\begin{aligned} &  \!\!\!\text {Structural model -- L2 (part 1):} \nonumber \\  &  x_{Bi}= \alpha _x + \zeta _{Bxi} \end{aligned}$$

4 $$\begin{aligned} &  y_{Bi} = \alpha _y + \zeta _{Byi} \end{aligned}$$

5 $$\begin{aligned} &  \beta _{yi} = \alpha _{\beta y} + \zeta _{\beta yi} \end{aligned}$$

Due to the latent decomposition of the predictor variable *x* into between- and within-person parts, the individual slopes $\beta _{yi}$ represent individual *i*’s WPA between sociality *x* and well-being *y* (Asparouhov & Muthén, 2019; Enders & Tofighi, 2007; Raudenbush & Bryk, 2002). The individual slopes vary around an average slope $\alpha _{\beta y}$ with a person-specific deviation $\zeta _{\beta yi}$, whose variability reflects interindividual differences in social reactivity. Similarly, the latent variable $x_{Bi}$ (person *i*’s average sociality) and the random intercept $y_{Bi}$ (person *i*’s average well-being) vary around the overall averages of sociality and well-being ($\alpha _x$ and $\alpha _y$), respectively, with person-specific deviations ($\zeta _{Bxi}$ and $\zeta _{Byi}$).

The expected (inverse) U-shaped association between social reactivity $\beta _{y}$ and life satisfaction *z* is modeled as a quadratic effect in a second part of the structural model on level 2:

6 $$\begin{aligned} &  \!\!\!\text {Structural model -- L2 (part 2):} \nonumber \\  &  z_{i} = b_0 + b_1\beta _{yi} + b_3\beta _{yi}^2 + \zeta _{zi} \end{aligned}$$

Here, the regression weights ($b_1$, $b_3$) are numbered nonconsecutively for consistency with a more general second-order polynomial model that we introduce for the WPA-congruence hypothesis below. The coefficient $b_3$ in Eq. 6 reflects the (inverse) U-shaped effect of interest.

The definition of the MSEM is completed by distributional assumptions for the latent variables on level 1 and level 2:

7 $$\begin{aligned} \text {L1: } \begin{bmatrix} x_{Wit} \\ \zeta _{Wyit} \end{bmatrix} \sim \mathcal {MVN}\left( \varvec{0}, \quad \begin{bmatrix} \sigma ^2_x & \\ 0 & \sigma ^2_y \\ \end{bmatrix} \right) = \mathcal {MVN} ( \varvec{0}, \Phi _W) \end{aligned}$$

8 $$\begin{aligned} \text {L2: } \begin{bmatrix} \zeta _{Bxi}\\ \zeta _{Byi} \\ \zeta _{\beta _yi} \\ \zeta _{zi} \end{bmatrix} \sim \mathcal {MVN}\left( \varvec{0}, \quad \begin{bmatrix} \tau ^2_x & \\ \tau _{xy} & \tau ^2_y \\ \tau _{x\beta _y} & \tau _{y\beta _y} & \tau ^2_{\beta _y} \\ \tau _{xz} & \tau _{yz} & 0 & \tau ^2_z \\ \end{bmatrix} \right) = \mathcal {MVN} ( \varvec{0}, \Phi _B) \end{aligned}$$

All of the nonzero terms in $\Phi _W$ and $\Phi _B$ can be estimated freely, whereas the others must be restricted to zero. The diagonal entries of $\Phi _W$ refer to the within-person variance of sociality ($\sigma ^2_x$) and the L1 residual variance of well-being ($\sigma ^2_y$). The diagonal of $\Phi _B$ contains the between-person variances of sociality ($\tau ^2_x$), well-being ($\tau ^2_y$), and social reactivity ($\tau ^2_{\beta _y}$) and the L2 residual variance of life satisfaction ($\tau ^2_z$). The covariance terms in $\Phi _B$ reflect between-person associations between these components, that is, between the measured variables, the random effects, and the L2 residuals. In practice, it is often reasonable to restrict some of these covariance terms in $\Phi _B$ (e.g., the conceptually least plausible ones) to zero, to simplify the estimation of the model (Bates, Kliegl, Vasishth, & Baayen, 2018).

### MSEM for a WPA-moderation hypothesis

A *WPA-moderation hypothesis* refers to the effect of an interaction between *two* WPAs on an outcome. For example, social reactivity may moderate the linear association between people’s stress reactivity – the WPA between situational stressfulness and negative affect – and life satisfaction. The data include $T_i$ assessments of sociality ($x_{it}$), well-being ($y_{it}$), stressfulness ($u_{it}$), and negative affect ($v_{it}$) for each individual *i*, and an L2 measure $z_i$ of life satisfaction.

The corresponding MSEM specifies an individual *i*’s social reactivity $\beta _{yi}$ and their stress reactivity $\beta _{vi}$ as individual slopes, extending the model above to a bivariate model:

9 $$\begin{aligned} &  \!\!\!\text {Measurement model (latent decomposition):} \nonumber \\  &  x_{it} = x_{Bi} + x_{Wit} \nonumber \\  &  u_{it} = u_{Bi} + u_{Wit} \end{aligned}$$

10 $$\begin{aligned} &  \!\!\!\text {Structural model -- L1: } \nonumber \\  &  y_{it} = y_{Bi} + \beta _{yi} x_{Wit} + \zeta _{Wyit} \nonumber \\  &  v_{it} = v_{Bi} + \beta _{vi} u_{Wit} + \zeta _{Wvit} \end{aligned}$$

11 $$\begin{aligned} &  \!\!\! \text {Structural model -- L2 (part 1): } \nonumber \\  &  x_{Bi} = \alpha _x + \zeta _{Bxi} \nonumber \\  &  u_{Bi} = \alpha _u + \zeta _{Bui} \end{aligned}$$

12 $$\begin{aligned} &  y_{Bi} = \alpha _y + \zeta _{Byi} \nonumber \\ &  v_{Bi} = \alpha _v + \zeta _{Bvi} \end{aligned}$$

13 $$\begin{aligned} &  \beta _{yi} = \alpha _{\beta y} + \zeta _{\beta yi} \nonumber \\ &  \beta _{vi} = \alpha _{\beta v} + \zeta _{\beta vi} \end{aligned}$$

Here, the interpretation of the model components is analogous to that of Eqs. 1 to 5. The main difference between the WPA-moderation and the WPA-U-shape model is that an interaction effect instead of a quadratic effect is now specified in the second part of the L2 structural model:

14 $$\begin{aligned} &  \!\!\!\text {Structural model -- L2 (part 2):} \nonumber \\  &  z_{i} = b_0 + b_1\beta _{yi} + b_2\beta _{vi} + b_4\beta _{yi}\beta _{vi} + \zeta _{zi} \end{aligned}$$

The coefficient $b_4$ of the interaction term reflects the potential moderation effect of interest. The model is again completed by distributional assumptions on both levels:

15 $$\begin{aligned} \text {L1: } \begin{bmatrix} x_{Wit} \\ u_{Wit} \\ \zeta _{Wyit} \\ \zeta _{Wvit} \end{bmatrix} \sim \mathcal {MVN}\left( \varvec{0}, \quad \begin{bmatrix} \sigma ^2_x & \\ \sigma _{xu} & \sigma ^2_u \\ 0 & 0 & \sigma ^2_y \\ 0 & 0 & \sigma _{yv} & \sigma ^2_v \\ \end{bmatrix} \right) = \mathcal {MVN} ( \varvec{0}, \Phi _W) \end{aligned}$$

16 $$\begin{aligned}&\text {L2: } \begin{bmatrix} \zeta _{Bxi}\\ \zeta _{Bui} \\ \zeta _{Byi} \\ \zeta _{Bvi} \\ \zeta _{\beta _yi} \\ \zeta _{\beta _vi} \\ \zeta _{zi} \end{bmatrix} \sim \mathcal {MVN}\left( \varvec{0}, \quad \begin{bmatrix} \tau ^2_x & \\ \tau _{xu} & \tau ^2_u \\ \tau _{xy} & \tau _{uy} & \tau ^2_y \\ \tau _{xv} & \tau _{uv} & \tau _{yv} & \tau ^2_v \\ \tau _{x\beta _y} & \tau _{u\beta _y} & \tau _{y\beta _y} & \tau _{v\beta _y} & \tau ^2_{\beta _y} \\ \tau _{x\beta _v} & \tau _{u\beta _v} & \tau _{y\beta _v} & \tau _{v\beta _v} & \tau _{\beta _y \beta _v} & \tau ^2_{\beta _v} \\ \tau _{xz} & \tau _{uz} & \tau _{yz} & \tau _{vz} & 0 & 0 & \tau ^2_z \\ \end{bmatrix} \right) \nonumber \\&\qquad \qquad \qquad \quad \!= \mathcal {MVN} ( \varvec{0}, \Phi _B) \end{aligned}$$

On level 1, the elements of $\Phi _W$ are the within-person variances of sociality ($\sigma ^2_x$) and situational stressfulness ($\sigma ^2_u$), the L1 residual variances ($\sigma ^2_y$, $\sigma ^2_v$), the L1 covariance between momentary sociality and stressfulness ($\sigma _{xu}$), and the covariance of the L1 residuals ($\sigma _{yv}$). On level 2, $\Phi _B$ contains the between-person variances of sociality ($\tau ^2_x$), stressfulness ($\tau ^2_u$), well-being ($\tau ^2_y$), negative affect ($\tau ^2_v$), social reactivity ($\tau ^2_{\beta _y}$), and stress reactivity ($\tau ^2_{\beta _v}$) and the L2 residual variance of life satisfaction ($\tau ^2_z$). The covariances involving a combination of *x*, *y*, $\beta _y$, and *z* are interpreted in the same way as described above for Eq. 8, and those involving a combination of *u*, *v*, $\beta _v$, and *z* have analogous interpretations referring to stressfulness, negative affect, and stress reactivity. In addition, $\Phi _B$ contains all pairwise covariances between the L2 parts of the social reactivity set of variables (people’s average sociality, well-being, social reactivity) and the stress reactivity variables (average stressfulness, negative affect, stress reactivity). Again, it will often be reasonable to set several of the covariances to zero to simplify the estimation of the model.

### MSEM for a WPA-congruence hypothesis

A *WPA-congruence hypothesis* also involves two WPAs. It posits that individuals (L2 units) with more congruent values of the two WPAs have higher (or lower) values on an outcome variable. As an example, assume that we expect positive effects of intrapersonal consistency in reactivities, in the sense that individuals whose level of social reactivity is similar to their level of stress reactivity have higher life satisfaction than people with more incongruent reactivities. An investigation of this hypothesis requires the same data as the WPA-moderation hypothesis, the same models for the two WPAs $\beta _{yi}$ and $\beta _{vi}$ (Eqs. 9 to 13), and identical distributional assumptions.

The L2 structural model that represents the WPA-congruence hypothesis is based on Response Surface Analysis (RSA; Box & Draper, 1987; Box & Wilson, 1951; Edwards & Parry, 1993). It is a second-order polynomial model in which life satisfaction *z* is predicted from the two slopes $\beta _{y}$ and $\beta _{v}$, their squared terms, and their interaction (see also Humberg et al. , in press):

17 $$\begin{aligned} &  \!\!\!\text {Structural model -- L2 (part 2):} \nonumber \\  &  z_{i} = b_0 + b_1\beta _{yi} + b_2\beta _{vi} + b_3\beta _{yi}^2 + b_4\beta _{yi}\beta _{vi} + b_5\beta _{vi}^2 + \zeta _{zi} \end{aligned}$$

The usual procedure of RSA involves using the regression weights $b_1, \dots , b_5$ to calculate six auxiliary parameters ($a_1, a_2, a_3, a_4, p_{10}, p_{11}$) that help interpret the model’s predictions. The model represents the congruence hypothesis if the parameters satisfy six respective conditions ($a_1=a_2=a_3=p_{10}=p_{11}-1=0, a_4<0$), which are typically checked with null hypothesis significance tests. For readers unfamiliar with RSA, we provide additional information about the auxiliary parameters in the supplement (https://osf.io/xucwf; for more detailed explanations, see, e.g., J. R. Edwards , 1993, 2002; Humberg, Nestler, & Back, 2019; Schönbrodt , 2016).

### Implementation and estimation

A major complexity of all three MSEM models (U-shape, moderation, congruence) is that their L2 structural models involve nonlinear functions of random slopes ($\beta _{yi}^2$, $\beta _{yi}\beta _{vi}$, $\beta _{vi}^2$). To date, it is possible to estimate such models only by using Bayesian analysis software, such as Mplus (versions 8 or newer; Asparouhov & Muthén, 2010a; Muthén & Muthén, 2017 see the code template at https://osf.io/xucwf), JAGS (Plummer, 2016), or Stan (Stan Development Team, 2022). These software programs employ MCMC algorithms with Gibbs and Metropolis-Hastings sampling techniques to sample the latent variables and their (potentially) nonlinear effects (Asparouhov & Muthén, 2010a, 2021; Gelman et al., 2013). For the case of *linear* WPA hypotheses, recent simulation research has shown that the MSEM approach performs well with regard to estimation accuracy, whereas it also tends toward high estimation uncertainty, especially when the numbers of time points and individuals are small (e.g., Liu & Rhemtulla, 2022; Neubauer et al. , 2022).

However, it is currently unclear whether these results generalize to the nonlinear case that we consider here. In addition, the application of MSEM in this manner currently requires either commercial software (Mplus) or significant familiarity with Bayesian software (e.g., JAGS, Stan), thus limiting the accessibility of the approach for applied researchers. Therefore, the first aim of this paper is to assess how well the MSEM approach performs in testing the three hypotheses (U-shape, moderation, congruence). The second aim is to compare it with alternative methods that avoid the complexity of the MSEM approach and its Bayesian estimation procedures. The alternative approaches can be implemented with only basic statistical methods and are described in more detail below.

## Alternatives to the MSEM approach

We considered three alternatives to MSEM, all of which consist of two separate computational steps. All three approaches require the same data as described above for the MSEM approach, that is, repeated measures of two L1 variables *x* and *y* (or four, with *u* and *v* as well, if two WPAs are of interest) and an L2 variable *z*.

### Simple two-step approach

The simple two-step approach separates the estimation of the individual slopes (Step 1) from the estimation of their nonlinear effects (Step 2). More specifically, in *Step 1*, estimates of individuals’ WPAs between *x* and *y* are obtained by estimating a multilevel random coefficients model, where individual *i*’s WPA is represented by the L1 slope $c_{yi}$ (e.g., Brose et al. , 2022; Neubauer et al. , 2020):

18 $$\begin{aligned} \text {Centering: } &  x^C_{it}&= x_{it} - \bar{x}_{i\bullet } \text { with } \bar{x}_{i\bullet } = \frac{1}{T_i} \sum _{t} x_{it} \end{aligned}$$

19 $$\begin{aligned} \text {Level 1: } &  y_{it}&= \iota _{yi} + c_{yi} x^C_{it} + R_{yit} \end{aligned}$$

20 $$\begin{aligned} \text {Level 2: } &  \iota _{yi}&= \gamma _{y00} + \gamma _{y01} \bar{x}_{i\bullet } + U_{y0i} \end{aligned}$$

21 $$\begin{aligned} &  c_{yi}&= \gamma _{y10} + U_{y1i} \end{aligned}$$

22 $$\begin{aligned} &  \text {with } R_{yit} \sim \mathcal {N}(0, {{\,\textrm{Var}\,}}(R_{yit})), \nonumber \\  &  \begin{bmatrix} U_{y0i} \\ U_{y1i} \end{bmatrix} \sim \mathcal {MVN} \left( \varvec{0}, \quad \begin{bmatrix} {{\,\textrm{Var}\,}}(U_{y0i}) & \\ {{\,\textrm{Cov}\,}}(U_{y0i},U_{y1i}) & {{\,\textrm{Var}\,}}(U_{y1i}) \\ \end{bmatrix} \right) \end{aligned}$$

Conceptually, this model’s specification of the individual slopes is similar to MSEM’s (Eqs. 1 to 5). One technical difference is that the L1 predictor *x* is centered at the (manifest) person means, thus implying that its between- and within-person components ($\bar{x}_{i\bullet }$ and $x^C_{it}$, respectively) represent manifest rather than latent variables. Moreover, the between-person association between *x* and *y* is not included as a covariance (as in $\Phi _B$ in MSEM) but as an L2 regression coefficient ($\gamma _{y01}$ in Eq. 20). The individual slope $c_{yi}$ defined in Eq. 19 represents the WPA between *x* and *y* for individual *i*. In the simple two-step approach, estimates $\hat{c}_{yi}$ of these slopes are obtained for each individual.

When the aim is to test a WPA-moderation or a WPA-congruence hypothesis, which involve two WPAs, estimates $\hat{c}_{vi}$ of $c_{vi}$, representing the WPA between *u* and *v*, are obtained with an analogous multilevel model:

23 $$\begin{aligned} \text {Centering: } &  u^C_{it}&= u_{it} - \bar{u}_{i\bullet } \text { with } \bar{u}_{i\bullet } = \frac{1}{T_i} \sum _{t} u_{it} \end{aligned}$$

24 $$\begin{aligned} \text {Level~1: } &  v_{it}&= \iota _{vi} + c_{vi} u^C_{it} + R_{vit} \end{aligned}$$

25 $$\begin{aligned} \text {Level~2: } &  \iota _{vi}&= \gamma _{v00} + \gamma _{v01} \bar{u}_{i\bullet } + U_{v0i} \end{aligned}$$

26 $$\begin{aligned} &  c_{vi}&= \gamma _{v10} + U_{v1i} \end{aligned}$$

27 $$\begin{aligned} &  \text {with } R_{vit} \sim \mathcal {N}(0, {{\,\textrm{Var}\,}}(R_{vit})), \nonumber \\  &  \begin{bmatrix} U_{v0i} \\ U_{v1i} \end{bmatrix} \sim \mathcal {MVN} \left( \varvec{0}, \quad \begin{bmatrix} {{\,\textrm{Var}\,}}(U_{v0i}) & \\ {{\,\textrm{Cov}\,}}(U_{v0i},U_{v1i}) & {{\,\textrm{Var}\,}}(U_{v1i}) \\ \end{bmatrix} \right) \end{aligned}$$

In *Step 2* of the simple two-step approach, the individual slope estimates $\hat{c}_{yi}$ and, if appropriate, $\hat{c}_{vi}$, are used as predictors in a regular regression model that tests the suggested nonlinear effect on *z*. For example, the most complex analysis model, the second-order polynomial for investigating a WPA-congruence hypothesis, uses the WPA estimates $\hat{c}_{y}$ and $\hat{c}_{v}$ and their squared and interaction terms to predict the outcome *z*:

28 $$\begin{aligned}&z_i = b_0 + b_1 \hat{c}_{yi} + b_2 \hat{c}_{vi} + b_3 \hat{c}_{yi}^2 + b_4 \hat{c}_{yi} \hat{c}_{vi} + b_5 \hat{c}_{vi}^2 + \varepsilon _i, \nonumber \\&\varepsilon _i \sim \mathcal {N}(0, {{\,\textrm{Var}\,}}(\varepsilon _i)) \end{aligned}$$

The models for the WPA-U-shape hypothesis (quadratic model: $b_2 = b_4 = b_5 = 0$) and the WPA-moderation hypothesis (interaction model: $b_3 = b_5 = 0$) are special cases of the second-order polynomial in Eq. 28. The interpretation of the model coefficients in terms of the hypotheses of interest is analogous to the interpretation of MSEM.

The simple two-step approach can be implemented, for example, in R with the *lme4* package (Bates, Mächler, Bolker, & Walker, 2015) in Step 1 and the *lm()* function in Step 2 (R Core Team , 2023, see the code template at https://osf.io/xucwf). To obtain estimates of the individual slopes ($\hat{c}_{yi}$ and, potentially, $\hat{c}_{vi}$) in Step 1, we use the expected a posteriori (EAP) estimates. In comparison with the alternative OLS estimate, one advantage of EAP estimates is that they include a reliability correction via shrinkage and therefore yield fewer parameter biases when they are used in a subsequent L2 model (Croon & van Veldhoven, 2007).^1^

Whereas the simple two-step approach is thereby straightforward to implement and also intuitive to understand (see also Cortina et al. , 2021), it has several limitations that originate from the fact that $\hat{c}_{yi}$ and $\hat{c}_{vi}$ are unreliable estimates of the (latent) individual slopes (Lüdtke et al., 2008; Neubauer et al., 2020; Raudenbush & Bryk, 2002); in fact, the first empirical investigations of psychologically relevant WPA constructs reported slope reliabilities around .5 even for studies with many measurement occasions (Kuper et al., 2022). The unreliability of the slopes is automatically taken into account by the MSEM approach because it uses their latent representations in the L2 analysis model of interest. By contrast, the simple two-step approach uses the estimated slopes as manifest variables in the L2 model and thereby falsely assumes that they are perfectly reliable. The use of EAP estimates of the slopes already represents a partial correction for unreliability, but prior work has shown that this partial correction is not always sufficient to ensure accurate estimates and inferences of their effects on L2 outcomes (Humberg et al., in press; Liu & Rhemtulla, 2022; Lüdtke et al., 2018). The SI and PV approaches that we describe below extend the simple two-step approach by implementing two different ways to take the unreliability of the slope estimates into account.

### Single-indicator (SI) approach

The general idea behind SI approaches is to avoid using the potentially unreliable estimates of interest (here: the estimated individual slopes) in the analysis model but instead use a latent representation thereof (Bollen, 1989; Hayduk, 1987). In the following, we adopt the SI approach described by Lai and Hsiao (2022), which is very general in that it contains several other SI approaches as special cases (e.g., fixed-reliability SI described by Savalei , 2019, see also the Discussion).

The first step of the SI approach is again to obtain EAP estimates of the individual slopes $\hat{c}_{yi}$ (and possibly $\hat{c}_{vi}$) for each individual *i* as in the simple two-step approach, while also estimating their standard errors $\hat{s}_{c_yi}$ (of $\hat{c}_{yi}$; and possibly $\hat{s}_{c_vi}$ of $\hat{c}_{vi}$), which correspond to the standard deviation of the slope’s conditional posterior distribution (e.g., Snijders & Berkhof, 2008). The estimated standard errors $\hat{s}_{c_yi}$ (and $\hat{s}_{c_vi}$) can vary across individuals, reflecting the fact that the reliabilities of individuals’ slope estimates can differ.

In the second step of the SI approach, a single-level SEM is used to estimate the nonlinear effect of interest. Its measurement model represents the individual slopes $\hat{c}_{y}$ as a latent variable $\eta _{y}$ with $\hat{c}_{y}$ as its only indicator (and analogous for $\hat{c}_{v}$). The factor variances, factor loadings, and error variances are defined as follows (Lai & Hsiao, 2022):

29 $$\begin{aligned} \begin{array}{ll} \text {Measurement model: } & \quad \\ \quad \hat{c}_{yi} = \lambda _{yi} \eta _{yi} + \xi _{yi} & \quad \hat{c}_{vi} = \lambda _{vi} \eta _{vi} + \xi _{vi}\\ \quad \text {Var}(\eta _{yi}) = 1 & \quad \text {Var}(\eta _{vi}) = 1 \\ \quad \lambda _{yi} = 1 - \hat{s}_{c_yi}^2 & \quad \lambda _{vi} = 1 - \hat{s}_{c_vi}^2 \\ \quad \xi _{yi} \!\sim \! \mathcal {N}(0, \hat{s}_{c_yi}^2 \cdot (1 \!-\! \hat{s}_{c_yi}^2) ) & \quad \xi _{vi} \!\sim \! \mathcal {N}(0, \hat{s}_{c_vi}^2 \cdot (1 \!-\! \hat{s}_{c_vi}^2) )\\ \end{array} \end{aligned}$$

The latent variable $\eta _{y}$ (and, if applicable, $\eta _{v}$) is then used in the structural model. For example, the second-order polynomial model used to test a WPA-congruence hypothesis is specified as follows:

30 $$\begin{aligned} &  \!\!\!\text {Structural model: } \nonumber \\  &  z_i = b_0 + b_1 \eta _{yi} + b_2 \eta _{vi} + b_3 \eta _{yi}^2 + b_4 \eta _{yi} \eta _{vi} + b_5 \eta _{vi}^2 + \varepsilon _i, \nonumber \\ &  \varepsilon _i \sim \mathcal {N}(0, {{\,\textrm{Var}\,}}(\varepsilon _i)) \end{aligned}$$

The WPA-U-shape hypothesis (quadratic model: $b_2 = b_4 = b_5 = 0$) and the WPA-moderation hypothesis (interaction model: $b_3 = b_5 = 0$) are represented by respective special cases of Eq. 30.

Whereas the first step of the SI approach can be implemented in R using the code for the simple two-step approach, estimating the SEM in the second step is challenging for two reasons: (a) the measurement models in Eq. 29 are person-specific and therefore need to be implemented with definition variables (Mehta & Neale, 2005) and (b) the structural model includes nonlinear effects of latent variables (e.g., $\eta _{yi}^2$). To our knowledge, estimating models with these two attributes is not (yet) possible with R. It can be achieved, for example, with Mplus, which uses maximum likelihood estimation with an EM algorithm for this purpose (see also Lai & Hsiao, 2022; see OpenMx as a potential alternative, Boker et al. , 2023; Neale et al. , 2016). Furthermore, due to the computational demand of estimating the person-specific model parameters, the estimation process can be computationally intensive. It can therefore be reasonable to simplify the model by setting the factor loadings and error variances to constant values: $\lambda _y = 1 - \overline{\hat{s}^2_{c_y\bullet }}$ and ${{\,\textrm{Var}\,}}(\xi _{yi}) = \overline{\hat{s}^2_{c_y\bullet }} \cdot (1 - \overline{\hat{s}^2_{c_y\bullet }})$, where $\overline{\hat{s}^2_{c_y\bullet }} = \frac{1}{P} \sum _{i} \hat{s}^2_{c_yi}$ is the average estimated variance of the slopes across individuals (see the code template at https://osf.io/xucwf). As Lai and Hsiao (2022) pointed out, the simplified SI approach is equivalent to approaches that have been investigated by, for example, Hsiao et al. (2018) and Savalei (2019). The only remaining challenge in the simplified SI approach then is the estimation of a single-level SEM with latent interaction terms, which can be achieved with a wider selection of software, including software for Bayesian analysis (e.g., Arminger & Muthén, 1998; Asparouhov & Muthén 2021) or latent moderated SEM (Klein & Moosbrugger, 2000; see also Sardeshmukh & Vandenberg, 2017).

### Plausible values (PV) approach

Plausible values (PV) approaches are usually discussed in the context of latent ability measurement as an alternative to using estimated person parameters in secondary analyses (Mislevy , 1991; see also Asparouhov & Muthén, 2010b; Lüdtke et al. , 2018). To account for unreliability, this method regards the individuals’ values on the latent variable of interest as missing data and generates multiple (e.g., $M=20$) so-called plausible values (PVs) for the latent variables for each individual by drawing them from their posterior predictive distribution given the observed data and a statistical model. The analysis model is then applied separately to each set of draws, and the results are pooled with Rubin’s rules (Rubin, 1987).

Here, we adapted the PV approach to the case in which the estimated person parameters are the individual slopes (e.g., $c_{yi}$). When these slopes are defined as random effects in a multilevel model (e.g., as in Eqs. 18 to 22), the person-specific posterior predictive distribution of each individual *i*’s slope $c_{yi}$ that is implied by the multilevel model is normal around $\hat{c}_{yi}$ with variance $\hat{s}^2_{c_yi}$ , provided that the estimates $\hat{c}_{yi}$ and $\hat{s}^2_{c_yi}$ have been obtained with an adequate (e.g., Bayesian) estimation procedure (Hoff, 2009). In addition, when drawing PVs, it is important to specify the multilevel model in such a way that it correctly reflects all associations that are present in the data, which – in our case – includes the nonlinear associations between the individual slopes and the L2 outcome variable (Asparouhov & Muthén, 2021; Goldstein, Carpenter, & Browne, 2014; Lüdtke et al., 2018; Mislevy, 1991; Monseur & Adams, 2009).

For the present purpose, however, we consider an implementation of the PV method that only approximates such an optimal PV approach without fully satisfying all of its requirements. The reason is that, for the complex task of modeling nonlinear effects of random slopes, the optimal PV approach would be more complex in terms of implementation and computation than the MSEM approach. By contrast, we focus on a simplified PV approach that attempts to strike a compromise between the benefits of PV and the simplicity of alternative approaches to MSEM and that can be implemented with only basic statistical methods.

Specifically, the first step of the PV approach considered here is to estimate a multilevel model that extends the one used in the simple two-step approach by including the L2 variable *z* as an L2 predictor of the random intercept and slope:

31 $$\begin{aligned} \text {Level 1: } &  y_{it}&= \iota '_{yi} + c'_{yi} x^C_{it} + R_{yit} \end{aligned}$$

32 $$\begin{aligned} \text {Level 2: } &  \iota '_{yi}&= \gamma '_{y00} + \gamma '_{y01} \bar{x}_{i\bullet } + \gamma '_{y02} z_i + \gamma '_{y03} \bar{x}_{i\bullet } z_i + U'_{y0i} \end{aligned}$$

33 $$\begin{aligned} &  c'_{yi}&= \gamma '_{y10} + \gamma '_{y10} z_i + U'_{y1i} \end{aligned}$$

The corresponding distributional assumptions are analogous to those stated in Eq. 22. We denote the parameters of this model with a prime (^′^) to distinguish them from those in Eqs 18 to 21. By using *z* as a predictor of the individual slopes $c'_{yi}$ in Eq. 33, the EAP estimate of an individual *i*’s slope ($\hat{c}'_{yi}$) and its standard error ($\hat{s}'_{c_yi}$) take into account a potential linear (but not a nonlinear) association between *z* and the slopes (Zyphur, Zhang, Preacher, & Bird, 2019). As a result of this first step, estimates of $\hat{c}'_{yi}$ and $\hat{s}'_{c_yi}$ are obtained for each individual *i*.

In a next step, for each individual *i*, a set of *M* plausible values $c_{yi(m)}$ for the individual’s slope are generated from the normal distribution

34 $$\begin{aligned} c_{yi(m)} \sim \mathcal {N} (\hat{c}'_{yi}, \hat{s}'^{2}_{c_yi} ), \ \text {for } m \in \{1,...,M\}, \end{aligned}$$

and similarly for $c_{vi}$. The analysis model (e.g., the second-order polynomial model in Eq. 28) is then estimated separately for each set of PVs, and the resulting estimates of the regression coefficients and standard errors are pooled using Rubin’s rules (Rubin, 1987).

This simplified PV approach can be implemented, for example, in R by estimating the multilevel model (Eqs. 31 to 33) with the *lme4* package (Bates et al., 2015) and the analysis model for each draw (e.g., Eq. 28) as a standard regression model. The simplified PV approach is thus easier to implement and estimate than the MSEM approach, and it addresses a central criticism of the simple two-step approach by taking the unreliability of the slope estimates into account via the variation in individuals’ slope draws. However, it has two limitations in comparison with an optimal implementation of the PV method. First, it ignores the uncertainty with which the parameters in the multilevel model are estimated. Second, the multilevel model that is used to obtain the PVs (Eqs. 31 to 33) includes only the linear relationship between the slopes and the L2 variable *z* while ignoring any nonlinear effects. One aim of our simulation study is to examine whether these limitations severely affect the performance of the approach or whether, by contrast, this version of the PV approach provides a reasonable, simpler alternative to the MSEM approach.

### Comparison of the approaches

We introduced four possible approaches for testing nonlinear WPA hypotheses (MSEM, simple two-step, SI, PV). In comparing these approaches from a practical perspective, the simple two-step approach is clearly the easiest to implement, as both steps can be conducted in R using basic multilevel and regression modeling. By contrast, it is more complex to implement SI and PV: The SI approach requires software that can estimate latent interaction terms in a single-level SEM, and the PV approach requires the generation and analysis of several draws of the individual slopes and thereby has more analytical steps than the simple two-step approach. The MSEM approach is the most computationally complex because it involves nonlinear functions of random slopes. Its estimation is thereby more time-consuming when compared with the other approaches, and it is currently possible only with a Bayesian approach as implemented in commercial software (Mplus) or in Bayesian statistical software (e.g., JAGS, Stan).

Comparing the approaches in terms of their model definitions, the simple two-step approach is based on the most restricted model. In comparison with the most complex MSEM approach, the simple two-step approach involves several (implicit) assumptions that prior research has shown to be problematic: First, the simple two-step approach does not take the unreliability of the individual slopes ($\hat{c}_{yi}$, $\hat{c}_{vi}$) into account when estimating the analysis model in Step 2, thereby implicitly assuming perfectly reliable slopes. This assumption is usually not met, which can lead to biased estimates and inferences (e.g., Bollen , 1989; Brose et al. , 2022; Cole & Preacher, 2014; Neubauer et al. , 2020; Westfall & Yarkoni, 2016); even more so given that the nonlinear combinations of the slopes used in the regression model are less reliable than the original slopes (Busemeyer & Jones, 1983). Second, a consequence of assuming perfectly reliable slopes is that the simple two-step approach implicitly assumes that there are no between-person differences in the slopes’ reliabilities, leading to heteroscedastic errors in the regression model when this assumption is not met (Lewis & Linzer, 2005). Third, the simple two-step approach uses a manifest decomposition of the L1 predictor variable(s); it therefore does not account for unreliability in the person means ($\bar{x}_{i\bullet }$ and $\bar{u}_{i\bullet }$; Grilli & Rampichini, 2011; Lüdtke et al. , 2008; Raudenbush & Bryk, 2002). Finally, because the slopes $\hat{c}_{y}$ and $\hat{c}_{v}$ are estimated in separate models, several of the covariances that can be freely estimated in MSEM (see Eqs. 15 and 16) are ignored in the simple two-step approach (e.g., the L1 residual covariance $\sigma _{yv}$; the L2 covariance $\tau _{yv}$). Ignoring these covariances may induce bias when they are in fact nonzero in the population.

Both the SI and PV approaches differ from the simple two-step approach in that they take into account the slopes’ imperfect reliabilities and between-person differences in these reliabilities. However, like the simple two-step approach, they also use a manifest decomposition of the predictors and restrict the same covariances to zero. The MSEM approach makes none of the mentioned assumptions and thus avoids introducing any potentially unjustified model constraints and the biases that may arise from them. At the same time, the fully latent nature of the MSEM approach can render its parameter estimates unstable under certain conditions, implying that the simpler approaches may even have advantages over MSEM in this regard (Humberg et al., in press; Lai & Hsiao, 2022; Ledgerwood & Shrout, 2011; Lüdtke et al., 2008; Savalei, 2019).

## Simulation study

We conducted a simulation study to evaluate the performance of the MSEM approach for modeling nonlinear effects of WPAs and compared it with the performances of the three simpler methods (two-step, SI, PV). To the best of our knowledge, these questions have only scarcely been examined before (see also Rohloff, Kohli, & Chung, 2022). Specifically, one study (Humberg et al., in press) compared the MSEM and the simple two-step approaches only for the WPA-congruence hypothesis. In this case, the simple two-step approach tended to underestimate the parameters and their standard errors, whereas MSEM yielded more accurate estimates and inferences but was also more prone to convergence problems and more variable estimates when the sample sizes on both levels were small. In the present simulation, we considerably extend this research by examining two further two-step approaches (SI and PV) and two additional classes of nonlinear effects of WPAs (U-shaped, moderation).

Specifically, in the simulation study, we varied the hypothesis of interest (U-shape, moderation, congruence). In each of the three corresponding conditions, we specified the relevant effect (e.g., U-shape) in the population model and estimated only the corresponding model (e.g., quadratic model) using the four approaches (MSEM, two-step, SI, PV). We also varied the strength of the respective nonlinear effect and the sample sizes on both levels of analysis (L1 $=$ time points, L2 $=$ individuals). We evaluated and compared the approaches on parameter estimation accuracy, inference accuracy, power, and false positive rates. At https://osf.io/xucwf, we provide code templates that implement the four approaches in the same way as in the simulation study (folder *code_templates*), and we provide the code for the study itself (folder *simulation_code*).

### Population model

We generated data for three variables $x_{it}, y_{it}, z_i$ (U-shape condition) or for five variables $x_{it}, y_{it}, u_{it}, v_{it}, z_{i}$ (moderation condition, congruence condition). Here, $i \in \{1, \dots , P\}$ denotes the L2 units (e.g., individuals) and $t \in \{1, \dots , T\}$ denotes the L1 units (time points). We considered $P = 200$ and 500 L2 units, representing typical and large sample sizes, respectively. Likewise, we considered $T = 15$ and 40 L1 measurements per individual (see Table 1). The four sample size conditions that resulted (each combination of $P = 200, 500$ and $T = 15, 40$) had balanced data, meaning that all individuals had L1 data for the same number of time points. In an additional sample size condition, we simulated unbalanced data, where the number of L1 measurements differed between L2 units. To this aim, we first generated data for $P = 200$ L2 units and $T = 40$ L1 measurements. Adopting the strategy and code from Neubauer et al. (2020), we then deleted varying numbers of L1 measurements for the different participants. The number of deleted values ranged from 0 to 60% (i.e., $T_i$ was between 16 and 40) and was drawn from a positively skewed distribution that yielded a typical pattern of unbalance (for more details, see https://osf.io/xucwf; Neubauer et al. , 2020). The results for the conditions with unbalanced data are reported after the results for the balanced data.

#### Population model: Level 1 structural model

We used the L1 and L2 models defined in Eqs. 9 to 16 as the data-generating model with the parameter values given in Table 1. We set all L1 variances to 1, and we set the L1 covariances to 0, except for the covariance of the L1 residuals ($\sigma _{yv}$), which was set to 0.2. All L2 intercepts were 0. The variances of the individual slopes were $\tau ^2_{\beta _y} = \tau ^2_{\beta _v} = 0.1$. The reliability of the slopes was thus .80 when $T = 40$, and it was .58 when $T=15$. The L2 variances $\tau ^2_x, \tau ^2_u, \tau ^2_y$, and $\tau ^2_v$ were set to values that implied ICCs of .5 for all variables. Most of the L2 covariance terms were set to 0. The exceptions were the L2 covariances $\tau _{xy}$, $\tau _{uv}$, and $\tau _{yv}$, the intercept-slope covariances $\tau _{y\beta _y}$ and $\tau _{v\beta _v}$, and the covariance of the individual slopes $\tau _{\beta _y \beta _v}$. These covariances were set to values that implied correlations of .20.

**Table 1:** Parameters of the population models

Parameter	Population values	Rationale
Sample sizes		
L2 sample size *P*	200 | 500	typical | large
L1 sample size *T*	15 | 40	typical | large
L1 structural model		
L1 variances $\sigma ^2_x, \sigma ^2_u$	1	
L1 residual variances $\sigma ^2_y, \sigma ^2_v$	1	
L1 residual covariance $\sigma _{yv}$	0.2	implies correlation $=$ .2
L1 all other covariances	0	
L2 all intercepts $\alpha$	0	yields centered variables
L2 variances $$\tau ^2_x, \tau ^2_u$$	1	implies $$ICC_x $$=$$ ICC_u $$= .5
L2 variances $$\tau ^2_y, \tau ^2_v$$	1.1	implies $$ICC_y $$=$$ ICC_v $$= .5
L2 variances $\tau ^2_{\beta _y}, \tau ^2_{\beta _v}$	0.1	implies ${{\,\textrm{Rel}\,}}(\beta _y)$=${{\,\textrm{Rel}\,}}(\beta _v)$= .80 when *T*=40
L2 residual variances $\tau ^2_z$	1	
L2 covariances $\tau _{xy}, \tau _{uv}$	0.21	implies correlations $=$ .2
L2 covariance $\tau _{yv}$	0.22	implies correlation $=$ .2
L2 covariances $\tau _{y\beta _y}, \tau _{v\beta _v}$	0.07	implies correlations $=$ .2
L2 covariance $\tau _{\beta _y\beta _v}$	0.02	implies correlation $=$ .2
L2 all other covariances	0	
L2 structural model		
*U-shape condition:*		
$b_3$	-4.1 | -2.2 | 0	$R^2$ $=$ .25 | .09 | 0
$b_2, b_4, b_5$	0	conformity with U-shape hypothesis
$b_0, b_1$	0	simplicity
*moderation condition:*		
$b_4$	-5.7 | -3.1 | 0	$R^2$ $=$ .25 | .09 | 0
$b_3, b_5$	0	conformity with moderation hypothesis
$b_0, b_1, b_2$	0	simplicity
*congruence condition:*		
$b_3$	-2.5 | -1.4 | 0	$R^2$ $=$ .25 | .09 | 0
$b_4$	$=$ $-2 b_3$	conformity with congruence hypothesis
$b_5$	$=$ $b_3$	conformity with congruence hypothesis
$b_0$	0	simplicity

*Note.* Rel $=$ reliability. The (co)variances in this table are the entries of $\Phi _W$ (Eq. 15) and $\Phi _B$ (Eq. 16). The coefficients of the L2 structural models refer to the global model $z = b_0 + b_1 \beta _y + b_2 \beta _v + b_3 \beta _y^2 + b_4 \beta _y \beta _v + b_5 \beta _v^2$ (Eq. 35)

#### Population model: Level 2 structural model

We generated the L2 outcome variable *z* from special cases of the second-order polynomial model:

35 $$\begin{aligned}&z= b_0 + b_1 \beta _y + b_2 \beta _v + b_3 \beta _y^2 + b_4 \beta _y \beta _v + b_5 \beta _v^2 + \zeta _z , \nonumber \\&\zeta _z \sim N(0, \tau _z) \end{aligned}$$

For the U-shape condition, we set $b_2$, $b_4$, and $b_5$ to zero to obtain the quadratic model (*U-shape model*), and we also set $b_0$ and $b_1$ to zero for simplicity. We varied the size $b_3$ of the quadratic effect as shown in Table 1. Specifically, we varied $b_3$ in three steps that corresponded to a certain amount of variance explained in the outcome *z*, namely, $R^2=.25$ (large effect), $R^2=.09$ (moderate effect), and $R^2=0$ (no effect). For the moderation condition, the corresponding *moderation model* is a special case of Eq. 35 with $b_3=b_5=0$. We set $b_0, b_1$, and $b_2$ to zero for simplicity, and we varied the size of the interaction effect $b_4$ to yield $R^2=.25, .09$, and 0 (see Table 1). Finally, for the congruence condition, we set the values of the coefficients to correspond with a *congruence model* (Schönbrodt, 2016), as shown in Table 1. In such a model, $b_1$ and $b_2$ are zero, $b_3$ and $b_5$ have the same (negative) value, and $b_4 = -2 b_3$, so that we only had to specify the value for $b_3$. We varied $b_3$ such that the variance explained by the congruence effect was $R^2=.25, .09$, and 0 (see Table 1).

In total, we considered $2 (P) \times 2 (T) \times 3 (hypothesis) \times 3 (R^2) = 36$ conditions with balanced data and $3 (hypothesis) \times 3 (R^2) = 9$ conditions in the unbalanced case. In each condition, we generated and analyzed 1,000 replications.

### Model estimation

In each of the three hypothesis conditions (U-shape, moderation, congruence), we estimated the correct model (quadratic, interaction, RSA) with each of the four approaches (MSEM, simple two-step, SI, PV).

#### MSEM approach

The parameters of the MSEM approach were estimated with Mplus (version 8, Muthén & Muthén, 2017; see the code templates at https://osf.io/xucwf). We specified the models as defined in Eqs. 1 to 17. To simplify the model, we set several of the covariances (Eqs. 15 and 16) to zero, all of which were also zero in the population (see Table 1). Specifically, we estimated the model with the following covariance structure (or a special case of it in the U-shape conditions involving only one WPA), where the bold zeroes refer to covariances that could be theoretically estimated but were set to zero:

36 $$\begin{aligned} \text {L1: } \Phi _W = {{\,\textrm{Cov}\,}}\left( \begin{bmatrix} x_{Wit} \\ u_{Wit} \\ \zeta _{Wyit} \\ \zeta _{Wvit} \end{bmatrix} \right) = \left( \begin{bmatrix} \sigma ^2_x & \\ \textbf{0} & \sigma ^2_u \\ 0 & 0 & \sigma ^2_y \\ 0 & 0 & \sigma _{yv} & \sigma ^2_v \\ \end{bmatrix} \right) \end{aligned}$$

37 $$\begin{aligned} \text {L2: } \Phi _B = {{\,\textrm{Cov}\,}}\left( \begin{bmatrix} \zeta _{Bxi}\\ \zeta _{Bui} \\ \zeta _{Byi} \\ \zeta _{Bvi} \\ \zeta _{\beta _yi} \\ \zeta _{\beta _vi} \\ \zeta _{zi} \end{bmatrix} \right) = \left( \begin{bmatrix} \tau ^2_x & \\ \textbf{0} & \tau ^2_u \\ \tau _{xy} & \textbf{0} & \tau ^2_y \\ \textbf{0} & \tau _{uv} & \tau _{yv} & \tau ^2_v \\ \textbf{0} & \textbf{0} & \tau _{y\beta _y} & \textbf{0} & \tau ^2_{\beta _y} \\ \textbf{0} & \textbf{0} & \textbf{0} & \tau _{v\beta _v} & \tau _{\beta _y \beta _v} & \tau ^2_{\beta _v} \\ \textbf{0} & \textbf{0} & \textbf{0} & \textbf{0} & 0 & 0 & \tau ^2_z \\ \end{bmatrix} \right) \end{aligned}$$

To estimate the models, we used the Bayes estimator with the default (flat) priors implemented in Mplus (Asparouhov & Muthén, 2010a). We used 120,000 MCMC iterations (half of which are discarded as burn-in) because a small presimulation showed that this was necessary to achieve convergence as indicated by effective sample sizes, trace plots, and potential scale reduction factors (Depaoli & van de Schoot, 2017; Gelman & Rubin, 1992; Zitzmann & Hecht, 2019). In the congruence condition, we also obtained estimates of the auxiliary RSA parameters ($a_1, ..., p_{11}$) with the model constraint option.

#### Simple two-step approach

We implemented the two-step approach in R (R Core Team , 2023; see the code template at https://osf.io/xucwf). We manually centered the L1 predictors $x_{it}$ and $u_{it}$ using their L2 means $\bar{x}_{i\bullet }$ and $\bar{u}_{i\bullet }$, estimated the multilevel models with the R package *lme4* (Bates et al., 2015), and extracted the EAP estimates of the individual slopes $\hat{c}_{yi}$ and $\hat{c}_{vi}$. The respective L2 analysis model (quadratic, interaction, or RSA model; e.g., Eq. 28) was estimated with the lm function. In the congruence condition, we computed estimates and standard errors for the auxiliary RSA parameters with the multivariate delta method implemented in the *car* package (Fox & Weisberg, 2019).

#### SI approach

For the SI approach, we used the multilevel model results from Step 1 of the simple two-step approach. Besides the EAP estimates of the individual slopes ($\hat{c}_{yi}$, $\hat{c}_{vi}$), we also extracted their conditional variances ($\hat{s}^2_{c_yi}$, $\hat{s}^2_{c_vi}$). We implemented the simplified version of the SI approach by aggregating the conditional variances across individuals. In the simulation conditions with balanced data, this simplification was in line with the simulated population (Lai & Hsiao, 2022) because all individuals in the simulation condition had the same number of time points and the same L1 residual variance, which implies that the population value of the slope reliability was the same for all individuals (Neubauer et al., 2020; Raudenbush & Bryk, 2002). We also used the simplified version of the SI approach in the conditions with unbalanced data, although the approach did not take the implied heterogeneity of the slope reliabilities into account (Lai & Hsiao, 2022; see also the Discussion section). To be consistent with the MSEM approach, we implemented Step 2 of the SI approach with the Bayesian estimator in Mplus (see the code template at https://osf.io/xucwf). We used the default priors and set the number of iterations to 12,000 (a tenth of the number in MSEM) because a presimulation showed that this number was sufficient for obtaining good convergence. Estimates of the auxiliary RSA parameters were obtained in the same way as in the MSEM approach.

#### PV approach

We implemented the PV approach in R (see the code template at https://osf.io/xucwf). In the first step, we computed the means, $\bar{x}_{i\bullet }$ and $\bar{u}_{i\bullet }$, estimated the multilevel model in Eqs. 31 to 33 (and, if appropriate, a respective model for the second slope $c'_{v}$) with the *lme4* package (Bates et al., 2015), extracted EAP estimates of the slopes ($\hat{c}'_{yi}$, $\hat{c}'_{vi}$) and also their conditional variances ($\hat{s}'^{2}_{c_yi}$, $\hat{s}'^{2}_{c_vi}$). For each individual and each of the two slopes, we drew $M=20$ plausible values as outlined above (see also Lüdtke et al. , 2018). Then, we estimated the analysis model separately for each set of PVs with the lm function and used Rubin’s rules to pool the results (Rubin , 1987; see also Enders , 2010). Standard errors of the auxiliary RSA parameters were obtained with the multivariate delta method (*car* package, Fox & Weisberg, 2019) and pooled in the same way.

### Evaluation criteria

We evaluated and compared the four approaches on parameter estimation accuracy, inference accuracy, power, and false positive rates. More specifically, first, we computed the *bias* for all regression coefficients ($b_1, ..., b_5$) in the respective analysis model as the difference between the average estimate and the true parameter value. For the parameters that were nonzero in the respective population model, we also computed the *relative bias* as the ratio of the bias and the true parameter value.

Second, we examined the estimation variability by inspecting the widths of the parameters’ empirical 95% confidence (two-step, PV) and credibility (MSEM, SI) intervals. To simplify the notation, we will use the abbreviation *CI* to refer to both types of intervals. For the two-step and PV approaches, the *empirical CI width* is given by ${{\,\mathrm{CI-width}\,}}_{emp}=2 \cdot z_{.975} \cdot s_{emp}$, where $s_{emp}$ is the empirical standard error. In MSEM and SI, the empirical CI width is computed as the range between the 2.5% and 97.5% quantiles of the sampling distribution.^2^ In addition, we computed the root-mean-squared error (*RMSE*) as the square root of the average squared difference between the estimate and the true parameter value, providing a measure that combines parameter bias and estimation variability.

Third, turning to the approaches’ quality of inference, we first calculated each parameter’s *estimated CI width* as the average width of its estimated CI across replications. We then calculated the *relative CI width differences*, $({{\,\mathrm{CI-width}\,}}_{est}-{{\,\mathrm{CI-width}\,}}_{emp})/{{\,\mathrm{CI-width}\,}}_{emp}$, to assess the accuracy of the approaches in estimating the estimation uncertainty. We also computed the *coverage rate* of a parameter as the percentage of CIs across simulation conditions that include the true parameter value.

Finally, in each of the three hypothesis conditions (U-shape, moderation, congruence), we inspected the *acceptance rates* of the respective hypothesis of interest. That is, we determined the proportion of cases per simulation condition in which the estimated coefficient of the respective nonlinear term ($b_3$ for U-shape, $b_4$ for moderation) was significant (95% CI excluded zero), or, in the congruence condition, in which the auxiliary parameters satisfied the six conditions that the RSA literature suggests for testing a congruence effect ($\hat{a}_1, \hat{a}_2, \hat{a}_3, \hat{p}_{10}$, and $\hat{p}_{11}-1$ not significantly different from zero, $\hat{a}_4$ significantly negative; e.g., see Edwards , 2002; Humberg et al. , 2019). The acceptance rate reflects the *power* to detect the respective nonlinear effect in conditions with $R^2=.25$ or $R^2=.09$, and the *false positive rate* when $R^2=0$.

### Convergence issues and run times

In the conditions with small sample sizes on both levels ($P=200, T=15$) and a model including one or more quadratic terms (U-shape model, congruence model), the MSEM model often failed to converge, meaning that even after 120,000 iterations, the potential scale reduction exceeded the cut-off that Mplus used for the respective model. In these cases, we redrew the respective data set. Overall, this happened 412 times out of the 6000 replications in the conditions in question ($P=200, T=15$, U-shape/congruence condition). The SI approach, which also uses a Bayesian estimator like MSEM, converged in 100% of the cases (after 12,000 iterations), including the cases in which MSEM did not converge.^3^ In a small follow-up simulation, we additionally explored the performance of the SI approach for cases in which MSEM did not converge. The results concerning the accuracy of the estimated parameters were comparable to the results reported below, whereas the SI approach tended to overestimate the widths of the CIs in samples that were problematic for MSEM. Therefore, we consider the SI approach a reasonable alternative when MSEM does not converge, whereas users should be aware that the significance tests tend to be conservative in such cases.

Moreover, we removed the CI width estimates for eight replications in the final MSEM results in conditions with the congruence model and the smallest sample sizes ($P=200, T=15$) because they were extreme outliers. For example, in one of these three replications, the CI width of $\hat{b}_5$ was 68,650 for this single case, whereas it ranged from 2 to 25 with a mean of 5.7 in the other cases from the same simulation condition.

The four approaches largely differed in the time they required for estimation. The simple two-step approach (0.06–0.07 s on average, depending on the sample size condition) and the PV approach (1.3–1.5 s) were the fastest approaches. The Bayesian estimation of the SI approach required between 40 s (when $P = 200$) and 100 s ($P = 500$). The MSEM approach had an average run time between 1 h ($P = 200$, $T = 15$) and 3 h ($P = 500$, $T = 40$) and was thereby clearly the approach that required the most time.

### Results for balanced data

#### Parameter estimates

Table 2 presents the biases of the regression weights $b_1$ to $b_5$ for all conditions with balanced data (all L2 units have the same number of L1 measurements). Biases printed in bold deviated from the true parameter value by more than 5%, and biases printed in italics and violet exceeded 10% (see https://osf.io/xucwf for a table with the relative biases).

For all approaches, the biases were almost always larger for parameters that were nonzero in the population, whereas parameters with a true value of zero were more accurately estimated. Considering the MSEM approach in the smallest sample size condition ($P=200, T=15$; first column of Table 2), MSEM estimates of the second-order term coefficients ($b_3, b_4, b_5$) were biased in some cases for all four hypothesis conditions, with relative biases up to 8% ($b_3$ in the congruence condition with $R^2=.25$). The direction of the biases was always away from zero, such that MSEM overestimated the strengths of some nonlinear effects. For the larger L1 sample size ($T=40$) and in all conditions with $P=500$, MSEM yielded approximately unbiased parameter estimates. Turning to the simpler approaches, the biases of the two-step and SI approaches seemed to depend on which hypothesis was considered. Both two-step and SI yielded approximately unbiased parameter estimates for the U-shape model for all sample sizes. Their estimates were thereby slightly more accurate than those of MSEM in the smallest sample size condition ($P=200, T=15$) in which the MSEM estimates were sometimes biased. For the moderation model, the two-step and SI approach provided approximately unbiased parameter estimates in all but the smallest sample size condition ($P=200, T=15$). In the smallest sample size condition ($P=200, T=15$), SI yielded a bias of slightly above 5% in one case, just like the MSEM approach, whereas the two-step approach was biased up to 7%. By contrast, for the congruence model, both the two-step and SI approaches underestimated the sizes of all nonlinear coefficients for all sample sizes, with about 10-20% bias in conditions with $T=15$ and around 10% bias when $T=40$. The two-step and SI approaches thereby estimated the congruence model with more bias than was the case for the MSEM approach. Finally, the PV approach tended to provide the most biased parameter estimates of all four approaches, throughout all simulation conditions. Specifically, this approach consistently underestimated the true parameter values (biased toward zero), with estimates that deviated from the true parameter values by 66–77% in the conditions with $T=15$ and by 35-49% in the conditions with $T=40$. The biases of two-step, SI, and PV varied only with the L1 sample size *T*, and thus with the reliability of the individual slopes, whereas they did not notably depend on the number of L2 units. The biases of the MSEM approach, by contrast, tended to decrease with increasing sample sizes on either level.

**Table 2:** Biases in the parameter estimates

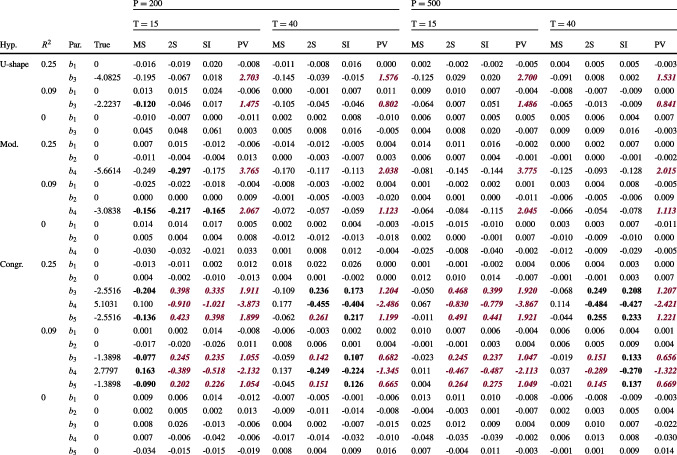		

*Note.* Hyp. $=$ hypothesis condition; U-shape $=$ U-shape condition; Mod. $=$ Moderation condition; Congr. $=$ Congruence condition; $R^2$ $=$ population value of $R^2$ of the L2 analysis model; Par. $=$ name of the parameter; True $=$ population value of the parameter; MS $=$ MSEM approach; 2S $=$ simple two-step approach; SI $=$ single-indicator approach; PV $=$ plausible values approach; *P* $=$ L2 sample size; *T* $=$ L1 sample size. The parameter labels $b_1$ to $b_5$ refer to Eq. 35. Biases are printed in bold when the absolute relative bias exceeded 5% of the true value and printed in italics and violet (dark gray) color when the relative bias exceeded 10%. See https://osf.io/xucwf for a table with the relative biases

**Table 3:** Empirical CI widths and relative CI width differences for two sample size conditions

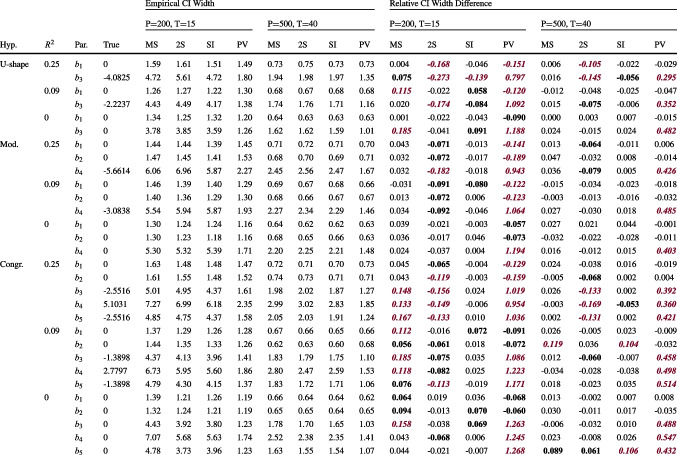		

*Note.* Hyp. $=$ hypothesis condition; U-shape $=$ U-shape condition; Mod. $=$ Moderation condition; Congr. $=$ Congruence condition; $R^2$ $=$ population value of $R^2$ of the L2 analysis model; Par. $=$ name of the parameter; True $=$ population value of the parameter; MS $=$ MSEM approach; 2S $=$ simple two-step approach; SI $=$ single-indicator approach; PV $=$ plausible values approach; *P* $=$ L2 sample size; *T* $=$ L1 sample size. The parameter labels $b_1$ to $b_5$ refer to Eq. 35. The relative CI width differences are computed by subtracting the empirical CI width per simulation condition from the average estimated CI width in this condition, divided by the empirical CI width. They are printed in bold when the absolute relative difference exceeded 5% and in italics and violet (dark gray) when it exceeded 10%

To summarize, the parameters of the U-shape model could be accurately estimated only with the simple two-step or SI approaches in the smallest sample size condition ($P=200, T=15$), and with MSEM, two-step, or SI for larger L1 or L2 sample sizes. The moderation model was almost accurately estimated by the MSEM and SI approach when both sample sizes were small, and again with MSEM, two-step, or SI for larger samples. By contrast, the most complex congruence model could be accurately estimated only by MSEM and only if the L1 or L2 sample size was not too small, whereas all other approaches yielded biased estimates. The PV approach consistently yielded large biases in all conditions.

#### Estimation variability

We inspected the empirical CI widths as a measure of estimation variability. The left half of Table 3 illustratively shows this criterion for the smallest and largest sample size conditions ($P=200, T=15$ and $P=500, T=40$; see https://osf.io/xucwf for all conditions). First comparing only MSEM, two-step, and SI (leaving the PV approach aside), the estimation variability for the parameters of the U-shape and moderation models tended to be smallest for MSEM and SI and larger for the two-step approach. For the congruence model, the variability tended to be smallest for the SI approach and largest for MSEM, with two-step in-between. With larger sample sizes, the empirical CI widths became very similar for MSEM, two-step, and SI. The RMSEs (reported in the supplement, see https://osf.io/xucwf) that balance this estimation variability with the accuracy of estimates exactly mimicked the pattern just described. In particular, the SI approach was among the approaches with the lowest RMSE in all conditions, whereby all differences in the RMSEs between the three approaches (MSEM, two-step, SI) were rather small and completely vanished in the largest sample size condition ($P=500, T=40$). The PV approach yielded the narrowest empirical CIs across all simulation conditions but the highest RMSEs (for the parameters $b_3, b_4$, and $b_5$ for the nonlinear terms), a result that likely came from the substantive parameter biases.

#### Parameter inference

The average widths of the estimated CIs for the parameters are reported in the supplement (https://osf.io/xucwf). A general pattern that occurred in almost all simulation conditions was that the estimated CI widths were largest for MSEM, followed by SI, then two-step, and finally the PV approach. Comparing the estimated and empirical CI widths (right half of Table 3), the CIs estimated with the MSEM approach tended to be wider than the respective empirical CIs, exceeding them by up to 18% for the congruence model in the smallest sample size condition ($P=200, T=15$). As the L2 sample size increased, and to a lesser extent as the L1 sample size increased, the CI estimates of MSEM became more accurate; but some CIs in the congruence model were still too wide (by up to 12%) even when both sample sizes were large ($P=500, T=40$). By contrast, the two-step approach consistently underestimated the CI widths, providing parameter estimates with overly narrow CIs (except for one case for which the CIs were too large in the congruence condition). These underestimations decreased as the L1 sample size increased but were not influenced by the L2 sample size. Even in the largest sample size condition ($P=500, T=40$), the two-step approach often underestimated the CI widths by more than 10%, with the bias tending to be strongest for the most complex congruence model (up to 17%). The SI approach yielded unsystematic discrepancies of up to 14% in estimating CI widths for some parameters for all sample sizes. The PV approach systematically and substantially overestimated the widths of the CIs for the parameters $b_3, b_4$, and $b_5$.

To summarize, the two-step (which underestimated the CI widths) and PV (which overestimated them) approaches clearly had the least accurate CI estimates. Comparing MSEM and SI, MSEM tended to provide more accurate estimates of the CI widths than SI for the U-shape and moderation models, particularly in conditions with $T=40$ or $P=500$. For the congruence model, SI yielded more accurate CI estimates than MSEM for samples with the smaller L2 sample size ($P=200$), whereas the two approaches’ CI estimates were on average similarly accurate for the larger L2 sample size ($P=500$).

The coverage rates (reported in the supplement, see https://osf.io/xucwf) are consistent with the results reported so far. They were most favorable for the MSEM approach, which yielded coverage rates in an acceptable range between 92.5% and 97.5% for all parameters and conditions. The two-step approach had consistent undercoverage (with a minimum of 80%) in all conditions, as a result of the CIs that were too narrow. The SI approach yielded undercoverage (minimum of 84%) in some of the cases in which either the parameter or its CI width was estimated with bias. The performance of the PV approach regarding coverage was again unacceptable.

#### Acceptance rates

Focusing on the MSEM, two-step, and SI approaches, first in terms of their power to detect the nonlinear effect in the population, MSEM was consistently the approach or was among the approaches with the highest power for all three hypotheses (see Table 4). The power of the simple two-step approach was similar to that of MSEM for the U-shape and moderation hypotheses. However, this high power was afforded by CIs that were too narrow for parameters that were nonzero in the population (see Table 3). For the congruence hypothesis, the power of the two-step approach was lower than that of the MSEM approach. The power of the SI approach was slightly lower than that of MSEM in the smallest sample size condition ($P=200, T=15$) for all hypotheses and was similar to that of MSEM when either of the sample sizes increased.

Considering the cases with $R^2=0$, for the U-shape and moderation hypotheses, the false positive rates for MSEM, two-step, and SI all varied unsystematically around the nominal level of 5%. For the congruence hypothesis, the false positive rates of the three approaches were much lower than 5% and increased as the sample sizes increased. This pattern could be expected due to the special nature of the hypothesis test, which involved significance tests of six parameters simultaneously. A comparison of the three approaches (MSEM, two-step, SI) showed no systematic differences in terms of falsely accepting the respective hypothesis.

The PV approach had the least favorable acceptance rates, as its power was usually much lower than the power of the other approaches, and it had false positive rates well below the nominal level of 5%. In the conditions with a congruence effect, the sometimes high power of almost 90% may seem like an advantage of the PV approach at first glance, but in fact, it reflects the approach’s violation of the chosen $\alpha$ level for the five auxiliary parameters ($\hat{a}_1, ..., \hat{p}_{11}-1$) that need to be nonsignificant in order to accept the congruence hypothesis.

### Results for unbalanced data

Table 5 presents the bias in the parameter estimates, the empirical CI widths, and the relative CI width differences for the conditions with unbalanced data in which the number of L2 units was $P=200$ and the number of L1 measurements $T_i$ ranged from 16 to 40 (for the remaining results, see https://osf.io/xucwf). Altogether the results indicated that all the approaches tended to perform better when the data were unbalanced than when the data were balanced with $P=200$ and $T=15$ but worse than when the data were balanced with $P=200$ and $T=40$. At the same time, the differences between the approaches were similar to the balanced case but favored the MSEM approach more when the data were unbalanced.

More specifically, the PV approach again yielded the least accurate parameter estimates and inferences as it did in the conditions with balanced data. For this reason, we now focus on comparing MSEM, two-step, and SI. An inspection of the biases (first three columns of Table 5) showed that the regression weight estimates provided by MSEM were approximately unbiased, whereas two-step and SI yielded accurate estimates for the U-shape and moderation models but 10–15% bias in estimating the parameters of the congruence model. This pattern was the same as in the balanced condition with $P=200$ and $T=40$.

Regarding estimation variability, the empirical CI widths (middle columns of Table 5) tended to be smallest for MSEM, followed by SI, and then the two-step approach. This result favored the MSEM approach more than when the data were balanced, where the variability in MSEM was similar to SI (U-shape and moderation models) or larger (congruence model).

Turning to parameter inferences, the average widths of the estimated CIs (reported at https://osf.io/xucwf) tended to be largest for the SI approach, followed by MSEM, and then the two-step approach. This pattern differed from the balanced case, where the CIs estimated with MSEM tended to be wider than those from the SI approach. Comparing the estimated and empirical CI widths (last three columns of Table 5), the nuanced differences that we reported for the balanced case (MSEM had more accurate CIs than SI for the U-shape and moderation models; vice versa for the congruence model) vanished when the data were unbalanced. Here, MSEM and SI had similar amounts of bias in estimating the widths of the CIs. The two-step approach tended to yield CIs that were too narrow by up to 18%, in line with its performance in the balanced case. The coverage rates (reported at https://osf.io/xucwf) of MSEM and SI were between 92.5% and 97.5% in all cases, whereas undercoverage occurred for the two-step approach as in the balanced case.

Finally, the power to detect an effect that was present in the population (see the last columns in Table 4) was highest for MSEM, as in the balanced case. However, in contrast to the balanced case, the power of two-step and SI to detect a nonlinear effect was about 8% lower than that of MSEM in the conditions with $R^2 = .09$.

### Discussion of the simulation results and recommendations

In the simulation study, we evaluated MSEM for testing nonlinear effects of WPAs and compared it with a simple two-step approach, an SI approach, and a PV approach. Overall, the MSEM approach performed well in testing the nonlinear WPA hypotheses that we considered. With the exception of conditions with sample sizes that were too small, MSEM yielded good performance in terms of its accuracy in estimating the parameters, coverage rates, and its likelihood of correctly detecting or rejecting the suggested nonlinear effects (consistent with, e.g., Humberg et al. , in press; Liu & Rhemtulla, 2022; Lüdtke et al. , 2008).

Concerning the alternative methods, our results imply that the SI approach qualifies as a viable alternative to MSEM, especially when only a small number of individuals (L2 units) participated in the study and when they provide (approximately) the same number of L1 assessments. In these conditions, SI and MSEM each had advantages when compared with each other. For example, for the U-shape model, SI yielded more accurate parameter estimates, but MSEM yielded more accurate CIs; this pattern was reversed for the congruence model. The differences between the approaches leveled out when it came to the final conclusion of the analysis, as the SI approach’s power to detect the nonlinear effect fell only slightly below that of MSEM. Moreover, when not only the L2, but also the L1 sample size was small, the SI approach outperformed MSEM in terms of its RMSEs for the U-shape and congruence model. When the L2 sample size was large, the MSEM approach outperformed SI in parameter accuracy for the congruence model and in the accuracy of the CIs of U-shaped and moderation effects. However, these advantages did not have any noticeable effect on the RMSE or acceptance rates. When the data were unbalanced, SI was as accurate as MSEM in estimating the coefficients of the U-shape and moderation models and in estimating the CIs for all three models. The only weakness in its performance relative to MSEM was that SI had about 8% less power to detect a nonlinear effect of medium strength.

**Table 4:** Acceptance rates of the respective hypothesis

		P $=$ 200								P $=$ 500											
		T $=$ 15				T $=$ 40				T $=$ 15				T $=$ 40				Unbalanced Data			
Hyp.	$R^2$	MS	2S	SI	PV	MS	2S	SI	PV	MS	2S	SI	PV	MS	2S	SI	PV	MS	2S	SI	PV
U-shape	0.25	96.4	95.6	95.7	34.1	99.8	99.7	99.7	96.6	100.0	100.0	100.0	89.5	100.0	100.0	100.0	100.0	99.7	98.8	98.7	85.9
	0.09	67.7	67.2	65.6	5.1	90.6	91.2	90.9	62.2	95.0	94.7	94.3	29.2	99.8	99.8	99.8	97.8	83.1	76.2	75.9	33.4
	0.00	3.9	5.2	4.7	0.1	6.0	5.7	5.9	0.2	4.9	5.5	4.7	0.0	4.9	4.9	4.5	0.4	4.7	5.2	4.5	0.4
Mod.	0.25	97.6	97.3	97.2	31.2	99.9	99.9	99.8	98.8	100.0	100.0	100.0	90.7	100.0	100.0	100.0	100.0	100.0	99.7	99.5	88.5
	0.09	67.2	67.3	64.9	3.7	90.1	90.3	89.5	63.6	96.9	95.9	96.5	28.6	100.0	100.0	100.0	99.4	85.8	78.9	77.7	36.5
	0.00	5.1	5.4	4.8	0.0	5.3	5.2	4.9	0.4	5.1	5.4	5.6	0.0	4.3	4.4	4.6	0.9	4.7	4.2	4.1	0.2
Congr.	0.25	79.7	67.7	76.7	13.6	83.0	77.8	82.5	82.1	79.4	72.3	77.1	59.5	81.5	75.8	80.7	88.4	82.2	74.0	82.0	64.7
	0.09	48.8	44.1	48.3	1.1	70.8	66.3	70.0	39.0	79.0	73.9	76.5	10.7	83.1	80.9	81.9	82.4	65.7	54.1	56.6	21.6
	0.00	1.1	1.6	1.5	0.0	2.3	2.3	2.1	0.0	1.5	1.6	1.2	0.0	2.1	2.1	2.0	0.1	1.4	1.6	1.4	0.0

*Note.* Hyp. $=$ hypothesis condition; U-shape $=$ U-shape condition; Mod. $=$ Moderation condition; Congr. $=$ Congruence condition; $R^2$ $=$ population value of $R^2$ of the L2 analysis model; MS $=$ MSEM approach; 2S $=$ simple two-step approach; SI $=$ single-indicator approach; PV $=$ plausible values approach; *P* $=$ L2 sample size; *T* $=$ L1 sample size

**Table 5:** Parameter biases, empirical CI widths, and relative CI width differences for conditions with unbalanced data

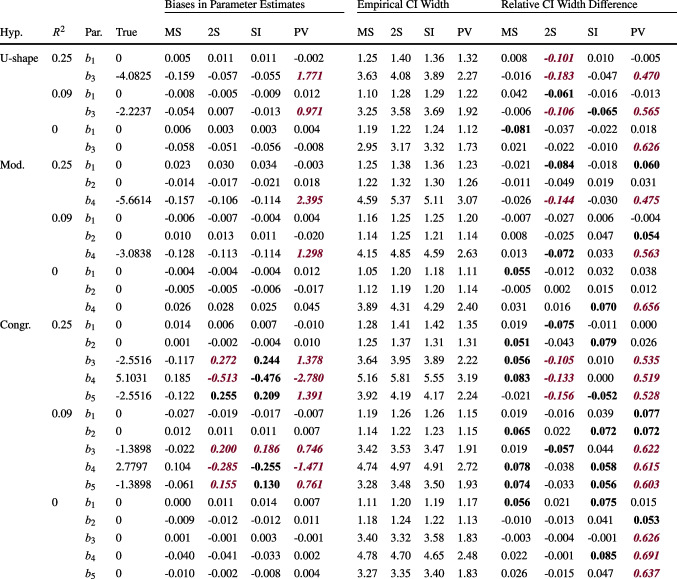		

*Note.* Hyp. $=$ hypothesis condition; U-shape $=$ U-shape condition; Mod. $=$ Moderation condition; Congr. $=$ Congruence condition; $R^2$ $=$ population value of $R^2$ of the L2 analysis model; MS $=$ MSEM approach; 2S $=$ simple two-step approach; SI $=$ single-indicator approach; PV $=$ plausible values approach

The good performance of the SI approach is particularly encouraging from a practical point of view because the approach is less computationally demanding and thus less time-consuming than MSEM. SI may also be beneficial in terms of its accessibility to researchers because the simplified version of the SI approach (without person-specific reliabilities) can be implemented in noncommercial software (e.g., R; see the Discussion).

Taken together, the SI approach appears to be an alternative that is equal to MSEM when the data are balanced and the L2 sample size is small. Moreover, even when the L2 sample size is large, we consider it justifiable to use SI instead of the slightly better performing MSEM approach when MSEM is not feasible due to the complexity of its estimation (e.g., convergence problems) or implementation (e.g., Mplus not affordable).

Moreover, extrapolating our results beyond the simulation conditions considered here, SI may even outperform the MSEM approach when L2 sample sizes are even smaller. Recent research has suggested replacing single-level SEM with SI in analyses of small samples (Lai & Hsiao, 2022; Savalei, 2019). Our study suggests that this principle might generalize to the testing of nonlinear effects of random slopes. The smallest L2 sample size that we considered here was $P=200$ L2 units, which is the upper boundary of what is categorized as a “small” sample in the literature (e.g., Savalei , 2019). Nonetheless, with this L2 sample size “at the boundary of small” (and for both L1 sample sizes), the SI approach outperformed MSEM in estimation accuracy for the U-shape model and in CI accuracy for the coefficients of the congruence model. Also, its power and false positive rates were similar to those of the MSEM approach (see also Kelcey , 2019; Lai & Hsiao, 2022; Savalei , 2019). It seems plausible to assume that the advantages of SI would be more pronounced for even smaller L2 samples – a conjecture that awaits confirmation in future studies. Also in line with prior studies, we found that in the small sample size conditions, none of the approaches yielded unbiased results overall; which emphasizes that the promising performance of SI should not be misconstrued as a justification to assess small samples, but as a glimpse of hope in situations that inhibit the recruitment of a large number of subjects (see also Zondervan-Zwijnenburg, Peeters, Depaoli, & vandeSchoot, 2017).

For the simple two-step approach, our results indicate that it systematically underestimated the width of all CIs for all population models, thereby suggesting a higher level of certainty in parameter estimation than was warranted. This finding is consistent with prior studies that have evaluated two-step approaches as alternatives to single- or multilevel SEMs (e.g., Ledgerwood & Shrout, 2011; Liu & Rhemtulla, 2022; Lüdtke et al. , 2018; Savalei , 2019; Westfall & Yarkoni, 2016). Overall, these findings underline that the simple two-step approach – despite being attractive from a practical perspective – should be avoided in practice (Brose et al., 2022; Cole & Preacher, 2014; Cortina et al., 2021; Savalei, 2019).

The simplified PV approach that we investigated here underestimated the coefficients of the nonlinear terms and overestimated their sampling variability, resulting in distorted acceptance rates in almost all conditions. One potential reason for the shortcomings of the PV approach was its simplified construction, which ignored estimation uncertainty and included only linear associations between the WPAs and the outcome. To investigate this possibility, we conducted additional simulations in which we (a) evaluated a fully nonlinear PV approach in which the PVs were generated directly on the basis of the MSEM model and (b) included conditions in which the population model included only linear effects (see https://osf.io/xucwf for these supplementary results). In both of these cases, each respective PV approach performed better than in our main simulation, but each tended to *under*estimate the widths of the parameters’ CIs and still tended to show inflated false positive rates. Future research could pursue these examinations, for example, by investigating tailored implementations of the PV approach and the effects of the parameters’ prior distributions (see Depaoli & Clifton, 2015; Holtmann, Koch, Lochner, & Eid, 2016; Zitzmann, Lüdtke, Robitzsch, & Marsh, 2016).

#### Recommendations

For applied researchers who aim to investigate nonlinear effects of WPAs, our first recommendation is to make an effort to assess large sample sizes at both levels, which can mean, for example, implementing ESM assessments with only a few items in order to increase participant commitment (Eisele et al. , 2022; but see also Hasselhorn, Ottenstein, & Lischetzke, 2022). If a large number of participants can be recruited (e.g., 500 or more), or if the participants differ essentially in their number of assessments, we recommend using the MSEM approach if Mplus is available or if the researcher is familiar enough with Bayesian statistical software to manually implement the model. If neither Mplus nor Bayesian software can be used (e.g., due to restricted financial resources), or if the MSEM model does not converge even with a large number of iterations, then researchers can instead implement the SI approach. By contrast, when the achievable sample size is small (e.g., 100 or less) and the data are approximately balanced, we recommend using the SI approach. This recommendation is based on our finding that SI’s performance was similar to MSEM’s in the smallest sample we considered (200) and on our conjecture that the small-sample benefits of SI reported in the literature (e.g., Lai & Hsiao 2022) will generalize to the modeling of nonlinear effects of random slopes. For “in between” sample sizes or amounts of balance, where it is unclear whether MSEM or SI performs best or where each approach can be expected to have its respective advantages (as in the balanced $P=200$ condition in our study), we recommend applying both approaches if possible. When they yield the same conclusion, the evidence found for the hypothesized nonlinear effect could then, also in the spirit of robustness analyses, be considered reliable.

## Empirical illustration

We illustrate the investigation of nonlinear effects of WPAs with combined data from two daily diary studies (see https://osf.io/xucwf for the data and code). Detailed descriptions of the study procedures and measures can be found in the study codebooks (https://osf.io/xucwf). The data we use here were provided by a total of $P=181$ individuals (143 female), who provided daily assessments for an average of $T=64$ time points ($min=19, max=82$). These L1 data were collected in daily assessments (on consecutive days in one study; over the course of four measurement waves in the other study).

We focus on a WPA-U-shape hypothesis positing an inverse U-shaped effect of social reactivity on life satisfaction. Here, social reactivity is operationalized as the WPA between repeated measures of sociality (“I spent a lot of time with other people today”; scale ranging from 1 = not at all to 6 = very; Schönbrodt & Gerstenberg, 2012) and a state measure of self-esteem (“I am satisfied with myself”; same scale from 1 to 6; Rosenberg , 1989). The L2 variable life satisfaction was measured with the satisfaction with life scale (Diener, Emmons, Larsen, & Griffin, 1985) in an online questionnaire at the beginning of the study.

**Table 6:** Results of the MSEM and SI approaches for the daily diary study

	MSEM		SI	
Parameter	Estimate	95%-CI	Estimate	95%-CI
$b_0$	**4.15**	[3.4, 4.72]	**4.21**	[3.58, 4.74]
$b_1$	**5.64**	[1.53, 12.21]	**5.14**	[1.46, 10.45]
$b_3$	**-10.81**	[-24.2, -2.42]	**-9.8**	[-19.93, -2.24]

*Note.* 95%-CI $=$ 95% credibility interval. Estimates of all parameters are printed in bold when the CI does not contain 0

Following the recommendations given above, we implemented both the MSEM and SI approaches for data analysis because the L2 sample size of $P=181$ was neither clearly large nor clearly small. Both approaches were implemented in the same way as in the simulation study, fitting a quadratic model on level 2.

**Fig. 1:**
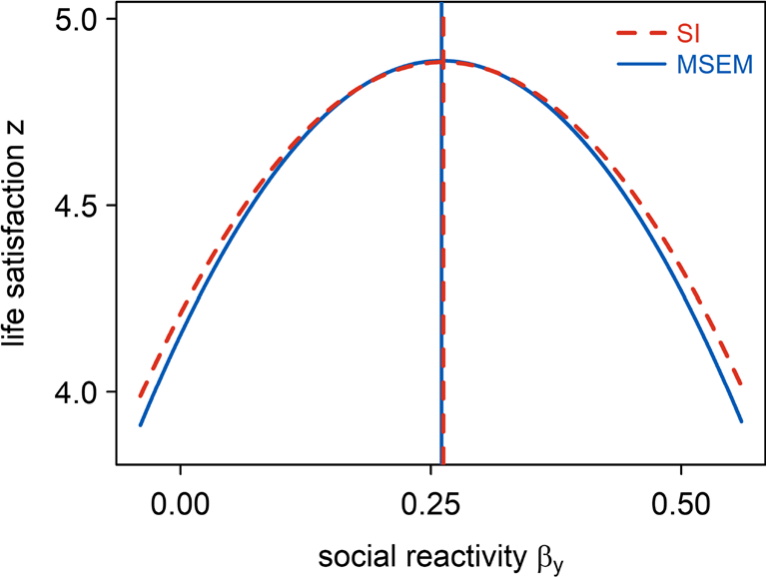
Graphs of the estimated quadratic models for the daily diary study

The parameters of the quadratic L2 model for both approaches are shown in Table 6 and visualized in Fig. 1. The $b_3$ coefficient from the quadratic term was significantly negative for both approaches, meaning that the association between social reactivity and life satisfaction had an inverted U-shape. For both approaches, the vertex of the inverted U was positioned at a slope value of $\beta _y=0.26$ ($=-b_1/2b_3$). The approaches differed only in the curvature $b_3$, which was stronger for MSEM than for SI (see Fig. 1 for a visualization). To reveal which parts of the inverted U could be interpreted in the final conclusion, we inspected the distribution of the EAP estimates of the individual slopes used in the SI approach. They were distributed around a mean of 0.24 with a standard deviation of 0.11, meaning that both “sides” of the inverted U may be interpreted. Overall, thus, both MSEM and SI point to an inverted U-shaped effect, such that people whose social reactivity is about average have the highest life satisfaction.

## Discussion

This article was concerned with evaluating statistical approaches that can be used to investigate nonlinear effects of random slopes, which are increasingly used in psychological research as measures of constructs that refer to the WPA between variables that fluctuate over time. Our research aims were to evaluate an MSEM approach and compare MSEM with three simpler approaches (simple two-step, SI, PV) that can also be used to investigate nonlinear effects of WPAs but that can more easily be implemented and estimated. In a nutshell, we found that the MSEM approach is a well-suited analytical strategy, at least for studies in which a large number of individuals participate. However, we also found that the SI approach can reasonably be recommended as an alternative approach, especially for balanced data with a small number of participants or when MSEM is unfeasible due to its complexity (e.g., when it does not converge). By contrast, the simple two-step approach and the PV approach cannot be recommended, as MSEM and SI outperformed them both.

A key finding of our simulation study is that the MSEM approach is well-suited for modeling nonlinear effects of random slopes. However, strictly speaking, we can infer this only for the specific implementation of MSEM considered in our simulation, in which many covariances were fixed to zero for simplification. We do not believe this decision affected the result of the simulation because we fixed the same covariances to zero in the population model. However, some of the covariance terms will not necessarily be zero in real data. Therefore, future research should explore (a) whether the MSEM approach produces biased results when the constrained covariances differ from zero in the population and (b) whether MSEM’s performance and its comparison with the simpler alternatives (especially SI) is affected when additional covariances are allowed in the model. Such work would also increase our understanding of how MSEM behaves in terms of the bias-variance trade-off, given that a model with a larger number of free covariances will fit the data more closely but is also more prone to convergence problems and larger estimation variabilities. In a similar vein, future research could explore whether the population variances of the L1 and L2 variables or the ICCs of the L1 variables have an impact on a method’s relative performance (see https://osf.io/xucwf for an additional simulation with variation in the ICCs).

Given the good performance of the SI approach in our simulation and respective prior findings for single-level SEMs (e.g., Lai & Hsiao, 2022; Savalei , 2019), we propose that it would be particularly interesting to conduct a more thorough comparison of the MSEM and SI approaches. For instance, future research could compare the two approaches for smaller sample sizes or in situations with more complex patterns of dependencies (e.g., correlated random slopes). Furthermore, other nonlinear population models (e.g., a violation of the congruence hypothesis due to shift or rotation of the model, see Humberg & Grund, 2022) should also be considered. Ultimately, the results of such studies may improve our recommendations regarding the choice between the two approaches. For example, we hope such results will help refine the sample size boundaries for choosing between MSEM and SI or develop tools to optimally allocate a given amount of financial resources to the necessary study investments (e.g., L1 sample size, L2 sample size, statistical software) in the interest of achieving the most reliable results (see also Zimmer, Henninger, & Debelak, 2022). Beyond comparing the SI approach to fully latent MSEM, it will also be relevant to compare it with two-step approaches that implement alternative reliability corrections for the random slopes. One promising approach would be Croon’s method (Croon, 2002). For the case of latent variables defined via multiple indicators in a measurement model, Croon’s correction has recently been adapted to multilevel models and shown to be a viable alternative to MSEM (Cox, Kelcey, & Bai, 2023; Devlieger & Rosseel, 2020; Kelcey, Cox, & Dong, 2019). Future research could adapt Croon’s method to models with latent interactions of random slopes and evaluate how well it performs in comparison with MSEM and SI.

Another important question for future studies refers to the performance of the methods when the data are unbalanced. In most longitudinal studies, the number of measurements varies between individuals, causing the reliabilities of the slopes to also differ across individuals. We considered one sample size condition with unbalanced data and found that the general pattern of results was similar to the balanced case but with MSEM outperforming the other approaches, including SI, in terms of the power to detect the nonlinear effects. However, we consider it important to continue to explore the differences between MSEM and SI in other unbalanced conditions and also to compare the different variants of the SI approach in such settings. In our simulation study, we implemented a version of the SI approach in which the person-specific estimation uncertainties of the slopes were averaged across all individuals, and this average was used to specify the factor loading and error variance in the single-indicator model on level 2. One alternative is to incorporate person-specific measurement models (Lai & Hsiao, 2022) that could be particularly beneficial when applied to unbalanced data. In the supplement, we report the results of this person-specific implementation of the SI approach in the simulated conditions with unbalanced data. Under the conditions considered here, the simplified SI approach *outperformed* the person-specific SI approach, especially in terms of overestimated sampling variability (consistent with results reported by Lai and Hsiao , 2022). However, we think that these observations require replication and deeper exploration in future studies. Moreover, a second alternative to simplified SI would be to simplify the approach even further than in our simulation study, by implementing a fixed-reliability approach in which reliabilities are not estimated from the data but are set to a fixed value (Savalei, 2019). Research has shown that this can stabilize the parameter estimates by removing the estimation step for the reliabilities (Oberski & Satorra, 2013; Savalei, 2019). However, the fixed-reliability SI approach can be used only if a good estimate (at least an upper bound) of the reliability is available (Savalei, 2019; Ulitzsch, Lüdtke, & Robitzsch, 2023), whereas applied research on WPAs is only just beginning to investigate their reliability (Kuper et al., 2022; Neubauer et al., 2020).

Furthermore, for consistency with the MSEM approach, we used a Bayesian approach to estimate the parameters of the SI approach here. However, it is also possible to estimate the SI parameters with other approaches that were suggested to estimate latent nonlinear effects (for guidance, see Cortina et al. , 2021), such as the latent moderated SEM approach (Klein & Moosbrugger, 2000; see also Sardeshmukh & Vandenberg, 2017) and a constrained or an unconstrained product indicator approach (Jaccard & Wan, 1995; Kenny & Judd, 1984; Marsh, Wen, & Hau, 2004). The main challenge in implementing the latter approach is in deriving the constraints of the product terms (i.e., the products of two random slopes). These derivations and a comparison of the different latent interaction approaches when combined with the SI approach seem to be interesting tasks for future research. In a similar vein, it would be worthwhile to evaluate the MSEM approach when using maximum likelihood for its estimation. However, this is currently not possible because it requires integration over a large number of random effects, and existing approaches are still restricted to the case of linear effects of random slopes (Nestler, 2020, 2021; Rockwood, 2020). A related question concerns the prior distributions. It may be possible that both the MSEM and SI approaches (when estimated with Bayes) would profit from using weakly informative prior distributions instead of the uninformative priors used here. When accurate prior information is available, so that the chosen priors are appropriate, their use could, for example, improve and stabilize the estimates in small samples (Depaoli & Clifton, 2015; Holtmann et al., 2016; Lai & Hsiao, 2022; Lüdtke et al., 2018; Ulitzsch et al., 2023; Zitzmann, Lüdtke, & Robitzsch, 2015). Lastly, all approaches examined in our simulation study are based on certain statistical assumptions (e.g., normally distributed data, homoscedastic residual terms) that do not have to be met in real data applications. It would therefore be interesting to examine how the approaches perform, for example, with nonnormal data (see Brandt, Umbach, Kelava, & Bollen, 2020 for corresponding insights in the context of single-level latent interaction models).

In addition to exploring different estimation methods, future research is also needed to better understand the influence of model complexity on the performances of the four approaches (see also Cole & Preacher, 2014). In our simulation study, we considered three L2 models with increasing complexity, namely, the quadratic model (one predictor, one nonlinear term), the interaction model (two predictors, one nonlinear term), and the RSA model (two predictors, three nonlinear terms). The patterns of results that we found for these three models indicate that model complexity affected how the approaches performed in nuanced ways. For example, the parameter bias monotonically increased as model complexity increased for all methods, but this increase in bias was steeper for the SI approach in comparison with MSEM. For example, SI outperformed MSEM in terms of bias for the quadratic model, whereas the opposite was true for the RSA model. Concerning the accuracy of the estimated CIs, model complexity did not appear to have any systematic effects. For example, the SI approach estimated the CIs of the quadratic model with more bias than the CIs of the more complex congruence model, whereas the MSEM estimates were similarly biased for these two models. Overall, these patterns indicate that it may be reasonable for applicants to tailor their choice of method (especially the choice between MSEM and SI) to the model considered, whereas the present findings do not support a conclusive recommendation for this choice. Future simulation studies should aim to unravel the mechanisms that underlie the nuanced differences and determine the extent to which they are driven by differences in the reliabilities of the nonlinear terms (e.g., quadratic terms are less reliable than interaction terms; MacCallum & Mar, 1995) or differences between how the methods represent the slope variability and the accuracy thereof. Beyond the three nonlinear models we considered in our simulation, the four approaches could also be evaluated for the simpler case of a linear model (for a comparison between MSEM and a simple two-step approach for this situation, see Liu & Rhemtulla, 2022). Although not the focus of the present study, we conducted an additional simulation for the linear model and report the results in the supplement (https://osf.io/xucwf). In short, the four approaches generally performed better in a linear than in a nonlinear model, and the differences between them were very similar to those in the U-shape model: MSEM performed well, and the SI approach was similar to MSEM in terms of estimation accuracy, variability, and power, but it provided CIs that were too narrow when the number of time points was small. In contrast to the main simulations, the simple two-step approach and the PV approach showed little bias in these conditions but again provided CIs that were too narrow. Future methodological work is needed to extend these initial results, particularly by conducting a more extensive comparison of MSEM and SI under varying conditions.

Finally, to go even one step further in terms of model complexity, it would also be interesting to apply the four approaches to models that incorporate measurement error into the assessed variables. Specifically, the models that we considered here treated the measured L1 (e.g., momentary well-being) and L2 (e.g., life satisfaction) variables as manifest, measurement-error-free variables. However, these variables will usually be not perfectly reliable, which can affect the results in undesired ways (Cole & Preacher, 2014). For example, if the L1 variables are measured with substantial measurement error, the random slopes will tend to be underestimated for all individuals. This underestimation can influence their variance across individuals and, consequently, the parameter estimates of the L2 model. As Lai and Hsiao (2022) pointed out, it would be interesting to investigate model extensions, particularly for the MSEM and SI approaches, which take this measurement error into account. This could be achieved, for example, by replacing the respective variable with a latent factor defined via multiple indicators (Rockwood, 2020) or by using a single-indicator factor model if the variable is measured with a single item with known reliability.

To sum up, our findings support the applicability of MSEM for testing nonlinear effects of random slopes, and they emphasize that single-indicator approaches are a promising alternative to MSEM, particularly in studies with few participants. Overall, both MSEM and SI seem to be valuable enrichments for the toolboxes of applied researchers, who can use our recommendations and templates to test theories that predict nonlinear effects of WPAs on relevant psychological outcomes.

## Data Availability

All data used in this article is available at https://osf.io/xucwf.

## Footnotes

1 In fact, in our simulation study, we also inspected the performance of a simple two-step approach using OLS instead of EAP estimates (see https://osf.io/xucwf for these additional results). However, it showed a comparatively inferior performance than the EAP version in terms of parameter biases and inferences, indicating that it cannot provide a preferable alternative to the two-step approach involving EAPs or to MSEM.

2 Although the Bayesian credibility intervals in MSEM and SI are not estimates of the 95% confidence intervals of the sampling distributions, we view the empirical CI width as providing a reasonable approximation of the estimation precision given that we used improper priors so that the Bayesian posterior distribution asymptotically converges toward the sampling distribution.

3 Because of a limitation in the implementation that we used to generate the first set of 500 replications, the comparison of MSEM and SI with respect to their convergence, as well as the statistics on computation times, was based on only 500 of the 1000 replications.

## Code Availability

All code is available at https://osf.io/xucwf.

## Declarations

### Author Note

Additional materials for this article can be found at https://osf.io/xucwf.

### Open Practices Statement

All code and data for the simulation study and the illustrative example analysis are available at https://osf.io/xucwf. This study was not preregistered.
